# MXene-Based Optical Fiber Sensors for Chemical and Biosensing: Review and Perspectives

**DOI:** 10.1021/acs.analchem.6c01831

**Published:** 2026-06-30

**Authors:** Chen Zhu, Huaijun Guan, Chenxi Huang, Rex E. Gerald II, Jie Huang

**Affiliations:** † Department of Electrical and Computer Engineering, 14717 Missouri University of Science and Technologya, Rolla, Missouri 65409, United States; ‡ Department of Chemistry, Missouri University of Science and Technology, Rolla, Missouri 65409, United States; § Silicon Engineering Group, Apple Inc., Cupertino, California 95014, United States

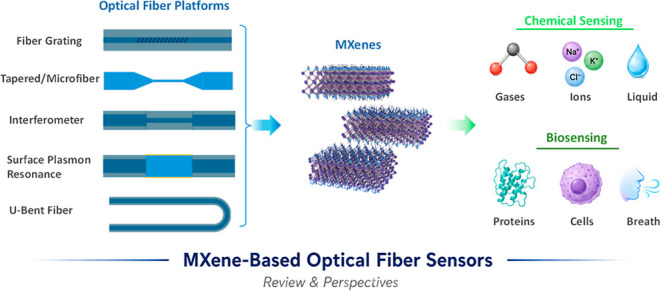

## Introduction

Sensors form the cornerstone of modern technological systems, underpinning applications ranging from environmental monitoring and industrial process control to biomedical diagnostics and human–machine interface devices. At the heart of every sensing system lies the sensing material, which transduces external stimuli, whether chemical, biological, or physical, into measurable signals.^1–3^ The intrinsic interaction between the material and the target stimulus ultimately determines key performance metrics, such as sensitivity, selectivity, response time, and long-term stability. Consequently, the development of advanced sensing materials has become a central pursuit in the next-generation sensor research. Over the past decades, a variety of functional materials have been synthesized and employed to construct high-performance sensors tailored for specific application domains. Among these, two-dimensional (2D) layered nanomaterials have attracted particular attention owing to their large surface-to-volume ratio, tunable electronic and optical structures, mechanical flexibility, and chemical versatility, which together enable superior interfacial coupling and transduction efficiency compared to their three-dimensional bulk counterparts.^4–7^

Among various families of 2D materials, such as graphene, transition-metal dichalcogenides, boron nitride, and black phosphorus,^8^ MXenes, a rapidly expanding class of transition-metal carbides and nitrides, have emerged as particularly promising candidates for sensing applications.^9^ First reported in 2011,^10^ MXenes are typically synthesized from layered MAX phases (M_*n*+1_AX_*n*_) through selective etching of the A-element. This top-down approach produces ultrathin sheets with the general formula M_*n*+1_X_*n*_T_*x*_, where M denotes an early transition metal, X represents carbon or nitrogen, and T refers to surface terminations such as −O, −OH, and −F.^11^ The combination of a large specific surface area, high electrical conductivity, and intrinsic hydrophilicity, together with abundant surface terminations that provide numerous active sites, endows MXenes with exceptional capability to interact with external stimuli through charge transfer, adsorption, or surface reactions. Moreover, their electronic, optical, and chemical properties can be flexibly tuned by compositional design, surface functionalization, and structural engineering, offering outstanding flexibility for material optimization. These synergistic attributes render MXenes highly effective transduction materials for various chemical and biological sensing applications.^12–16^

Over the past few years, MXene-based sensors have been demonstrated for detecting a wide variety of physical, chemical, and biological quantities through four fundamental transduction mechanisms: electrical, electrochemical, structural, and optical. Several comprehensive reviews have summarized these developments, highlighting the performance metrics, design strategies, and application domains. Early overviews categorized MXene sensors into chemical, biological, and physical types, emphasizing their high electrical conductivity and rich surface chemistry as key enablers of enhanced sensitivity and selectivity.^14^ Subsequent reports focused primarily on Ti_3_C_2_T_*x*_-based sensors, elucidating design principles for charge transfer interfaces and electrochemical reactions.^12^ More recent efforts have investigated MXene composites with graphene, metal oxides, or polymers for gas detection as well as MXene-based electrical and optical transducers for biosensing and intelligent devices.^16,17^ These studies establish MXenes as powerful material platforms for advanced sensing. Nevertheless, most existing work has concentrated on planar electrical and electrochemical configurations, while optical transduction mechanisms, particularly those realized on optical fiber platforms, remain comparatively underexplored despite their distinct advantages for remote, localized, multiplexed, and label-free chemical and biological sensing.

Over the past two decades, optical fiber sensors have experienced tremendous growth and advancement, evolving from simple point sensors for basic physical measurements (e.g., temperature and strain) into distributed and multiplexed systems capable of monitoring complex chemical and biological parameters, including clinically relevant biomarkers.^18–20^ Their miniature size, mechanical flexibility, and ability to operate over long distances have established optical fibers as one of the most versatile sensing platforms in modern science and engineering. In particular, optical fiber sensors provide an inherently compatible framework for leveraging the electronic and photonic characteristics of MXenes. Various fiber geometries, such as tapered, D-shaped, and microstructured fibers, generate strong evanescent fields that extend into the surrounding medium, allowing surface-bound materials to modulate the transmitted or reflected optical signal.^21–24^ When MXenes are deposited or integrated onto these regions, their high refractive index (RI), broadband absorption, and tunable optical constants induce pronounced spectral or intensity variations in response to target analytes. In interferometric configurations such as Mach–Zehnder or modal interferometers,^25,26^ MXene layers can modify the effective RI or optical phase, translating interfacial chemical or biochemical reactions into measurable fringe shifts. Likewise, the metallic conductivity of MXenes supports surface plasmon excitation, enabling highly sensitive RI sensing in the visible and near-infrared regions.^27^ Beyond single-point sensing, MXene coatings can also be incorporated into grating-based fiber architectures, potentially facilitating compact, multiplexed, and real-time optical sensor networks for advanced chemical and biological detection.

Despite these promising prospects, the understanding of MXene–fiber hybrid architectures remains limited. Previous reviews on MXene sensors have mainly focused on general material properties or electrical and electrochemical mechanisms,^28–30^ whereas optical fiber integration introduces additional factors such as film uniformity and thickness control, light-coupling efficiency, and adhesion to silica cores or claddings. Addressing these issues is essential to fully unlock the potential of MXene-functionalized optical fibers for practical chemical and biosensing applications. To bridge this gap, this review presents an overview of MXene-based optical fiber sensors for chemical and biological detection, with an emphasis on sensing mechanisms, integration strategies, and performance enhancement. We first outline the optical transduction principles of different fiber configurations, including evanescent-field, interferometric, grating-based, and surface plasmon resonance (SPR) architectures, and then summarize the key material and optical properties of MXenes that are relevant to light–matter interaction and interfacial sensing. The discussion also highlights how MXenes enhance the light–matter coupling and sensing performance in these configurations. We then review recent demonstrations of MXene-functionalized optical fibers. Finally, we discuss current challenges and future opportunities, including oxidation resistance, film adhesion, reproducibility, and prospects for multifunctional and distributed sensing enabled by hybrid photonic and plasmonic effects. Overall, this review aims to connect MXene materials science with fiber optics and provide design guidance for developing high-performance and scalable optical fiber chemical and biosensors.

## Optical Fiber Sensing Principles and MXene Integration

Originally developed for long-distance, low-loss telecommunications, optical fibers have evolved into a powerful platform for sensing applications. Optical fiber sensors have been successfully deployed in a wide range of fields, including structural health monitoring, downhole and environmental monitoring, and chemical and biological detection.^18,31,32^ Among various fiber sensing modalities, RI sensors have drawn particular attention for chemical and biosensing because of their compact size, high sensitivity, immunity to electromagnetic interference, capability for remote operation, and potential for multiplexed and distributed measurements. The operating principle of optical fiber RI sensors relies on light–matter interaction, where variations in the RI of the surrounding medium influence the properties of the guided light, such as intensity, wavelength, phase, or polarization, through the fiber’s evanescent field. In recent years, efforts to improve the sensitivity of optical fiber sensors have increasingly focused on functional material integration, where the coating layer on the fiber determines the efficiency of light–matter interaction. Among the wide variety of materials investigated, 2D nanomaterials have shown particular promise due to their large surface area, tunable optical and electronic properties, and strong surface reactivity. Within this class, MXenes have emerged as especially attractive candidates for optical fiber sensing. MXenes exhibit high electrical conductivity, excellent hydrophilicity, large specific surface area, and abundant surface functional groups, making them attractive candidates for a wide range of sensing technologies. In the context of optical fiber sensing, these attributes work synergistically with the intrinsic optical properties of MXenes, which determine how they interact with light and respond to surrounding chemical or biological stimuli at the fiber–material interface. In this section, we review the major optical fiber sensing principles that have been integrated with MXenes for sensing applications, including interferometric, evanescent-field-based, grating-based, and SPR configurations, and discuss how MXenes enhance sensor performance through improved light–matter coupling, surface reactivity, and optical tunability. Figure 1 shows an overview of recently developed MXene-enhanced optical fiber sensors.

**Figure 1:**
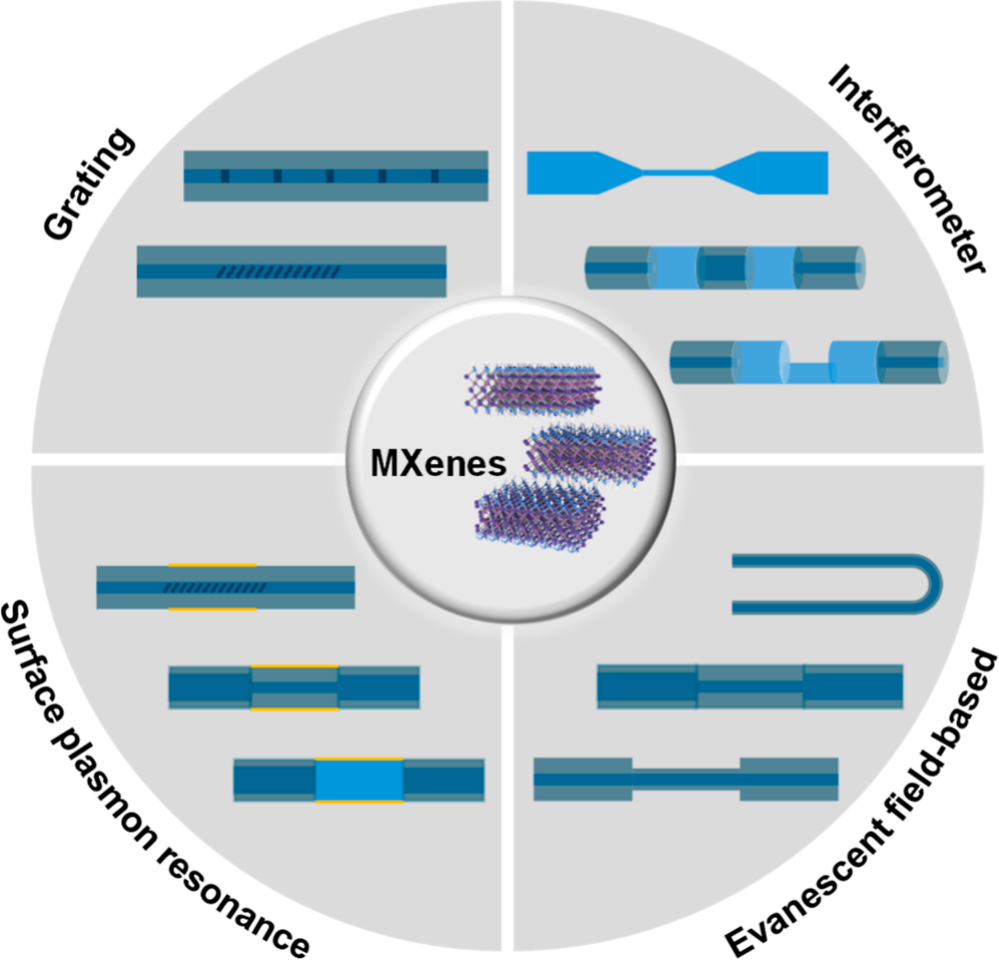
Schematic overview of representative optical fiber sensing architectures incorporating MXene functional layers and their associated transduction mechanisms. The illustrated configurations highlight how MXenes are integrated with different fiber platforms—including interferometric fibers, fiber Bragg gratings, surface plasmon resonance, and evanescent-field-based waveguides—to enhance light–matter interaction at the sensing interface. In each case, the MXene layer serves a dual role: (i) concentrating target analytes at the fiber surface through adsorption and (ii) modifying local optical and electromagnetic boundary conditions via changes in refractive index, absorption, and electronic conductivity. These adsorption-induced changes alter key optical parameters such as effective mode index, propagation loss, phase, or plasmonic resonance conditions, resulting in measurable shifts in wavelength, intensity, or spectral line shape. The figure illustrates the versatility of MXenes as active transduction layers across multiple fiber geometries and sensing modalities, emphasizing their ability to couple surface chemistry directly to optical signal modulation.

## Interferometric Sensing

Optical fiber interferometers are among the most widely used sensing configurations owing to their high sensitivity and versatile design. Various interferometric architectures, including the Michelson interferometer (MI), Mach–Zehnder interferometer (MZI), Sagnac interferometer (SI), Fabry–Perot interferometer (FPI), and modal interferometer, have been demonstrated for the measurement of a broad range of physical, chemical, and biological quantities.^33^ The fundamental operating principle of an interferometer in sensing applications is to convert the parameter of interest, such as strain, temperature, or analyte concentration, into a measurable phase delay or optical path difference between two interfering light beams. Changes in the phase or optical path manifest as shifts in the interference fringes, which can be accurately monitored through spectral or intensity interrogation. Considering a simple two-beam interferometer, where one beam propagates through the sensing arm and the other through the reference arm, the resulting transmission or reflection spectrum, i.e., the interference pattern, can be expressed as

1 $$I=I_{sensing}+I_{reference}+2\sqrt{I_{sensing}I_{reference}}\cos\left(\phi \right)$$

where *I*_sensing_ and *I*_reference_ denote the light intensities traveling through the sensing and reference paths, respectively, and ϕ represents the phase difference between the two arms. The phase difference can be written as

2 $$\phi =\frac{2\pi \Delta n_{eff}l}{\lambda }$$

where λ is the optical wavelength of the probe light, *l* is the length difference between the two arms of the interferometer, and Δ*n*_eff_ is the effective RI difference between them. When the phase-matching condition is satisfied, a series of local minima (or dips) appear in the transmission/reflection spectrum. The dip wavelengths can be expressed as

3 $$\lambda_{dip}=\frac{2\Delta n_{eff}L}{2k+1}$$

where *k* is an integer denoting the interference order. Therefore, in an optical fiber interferometer-based RI sensor, variations in the surrounding medium alter the effective RI difference between the two arms of the interferometer, resulting in measurable spectral shifts or intensity variations across different wavelengths.

### Microfiber-Based Interferometric Sensors

Figure 2 summarizes the optical fiber interferometer configurations that have been utilized in the development of MXene-based sensors, including microfiber devices, MZIs, Fabry–Perot cavities, and modal interferometers. As shown in Figure 2a, the microfiber-based device is one of the simplest configurations for RI sensing.^34^ By tapering a section of a standard single-mode fiber (SMF), the evanescent field of the guided light can be readily accessed. A tapered optical fiber (or microfiber) consists of a uniform waist region with a reduced diameter that is connected to two conical transition sections, where the fiber diameter gradually changes to merge with the unmodified SMF. The tapering process is typically achieved by stretching an SMF using two computer-controlled translation stages while locally heating it with a flame or a CO_2_ laser. The sensing performance of a tapered fiber is primarily influenced by the geometry of the conical regions and the diameter of the taper waist. In general, a smaller waist diameter yields a stronger evanescent field and, consequently, a stronger interaction with the surrounding medium, leading to higher RI sensitivity. Tapered fibers are commonly categorized as adiabatic or nonadiabatic, depending on the transition angle of the taper region. In adiabatic tapers, the gradual transition preserves cylindrical symmetry, and optical power remains confined within the fundamental mode. In nonadiabatic tapers, however, the abrupt transition causes mode coupling between the fundamental and higher-order modes. For example, when the waist diameter is less than 10 μm, the fundamental LP_01_ mode can couple into HE_11_ and HE_12_ modes. Two main approaches are used to quantify the interaction between the evanescent field and the external environment. The first involves monitoring the attenuation of transmitted light intensity through the tapered region, which directly reflects absorption or scattering losses in the surrounding medium. The second approach exploits the interferometric response arising from effective RI changes in the propagating modes. In a nonadiabatic taper with a waist diameter of 5.2 μm, variations in the surrounding medium modify the effective RI difference between the HE_11_ and HE_12_ modes, producing a measurable spectral shift in the interference fringes of the output spectrum.^35^

**Figure 2:**
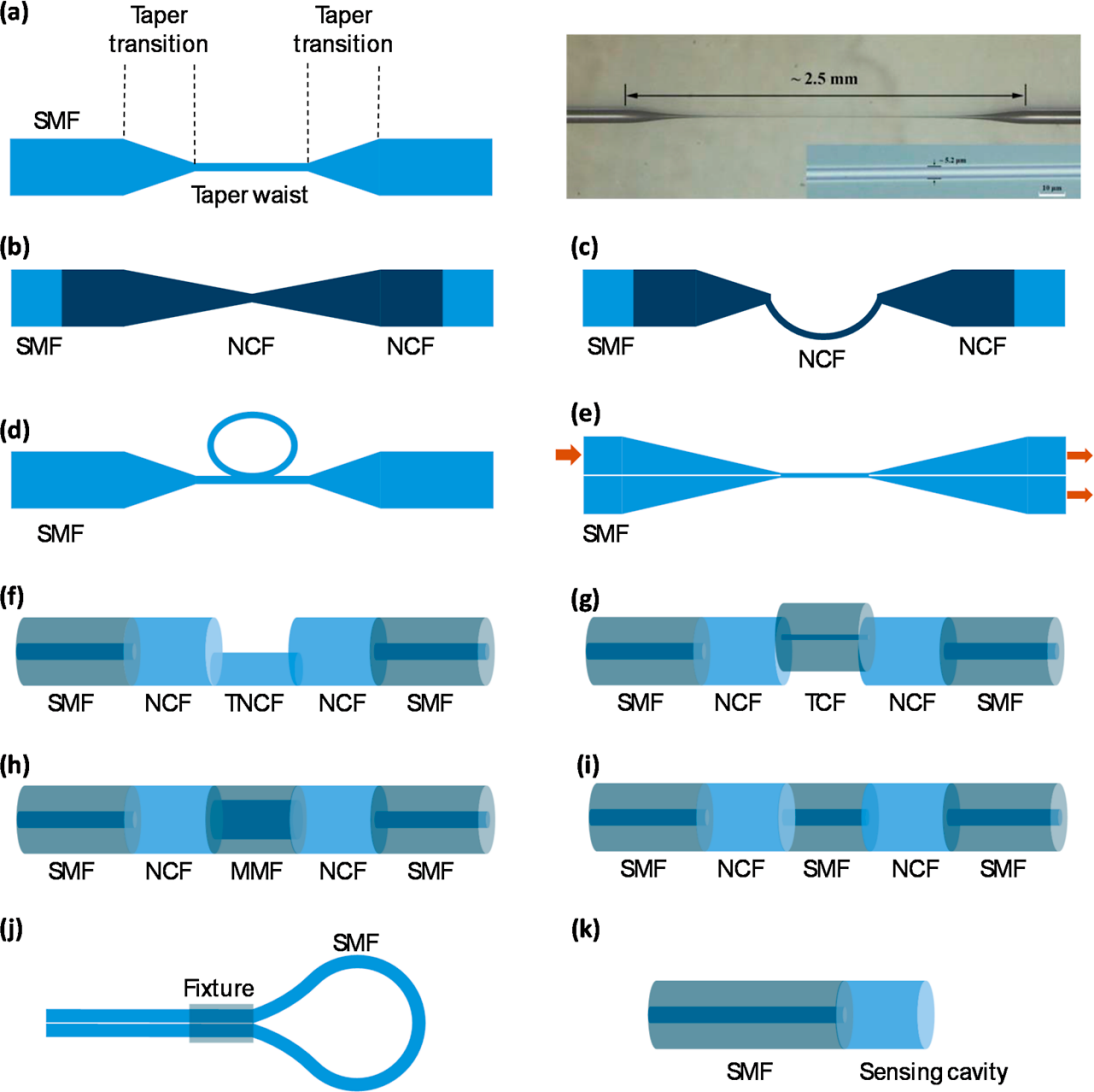
Summary of representative interferometer configurations integrated with MXenes for sensor development. (a) Schematic and photograph of a tapered microfiber interferometer. Adapted with permission from ref 35. Copyright 2022 IEEE. (b) Tapered no-core fiber (NCF) interferometer. (c) U-shaped tapered NCF interferometer. (d) Microfiber knot resonator (MKR). (e) Optical microfiber coupler (OMC) interferometer. (f) Composite interferometer consisting of single-mode fiber (SMF), NCF, thin NCF, NCF, and SMF. (g) Composite interferometer consisting of SMF, NCF, thin-core fiber (TCF), NCF, and SMF. (h) Composite interferometer consisting of SMF, NCF, multimode fiber (MMF), NCF, and SMF. (i) Composite interferometer consisting of SMF, NCF, SMF, NCF, and SMF. (j) Balloon-shaped fiber modal interferometer. (k) Fabry–Perot interferometer (FPI).

A slight variation of the microfiber interferometer is the tapered no-core fiber (TNCF) device, as shown in Figure 2b.^36^ In this configuration, a section of NCF is fusion spliced between two SMFs and subsequently tapered to a waist diameter of approximately 11.6 μm. Rather than relying solely on extreme tapering to achieve mode coupling, the core diameter mismatch between the SMF and the NCF excites higher-order modes that interfere with the fundamental mode, thereby producing a stable interference pattern at the output. Changes surrounding the TNCF can thus be read out by monitoring the interference pattern. To further increase the sensitivity, the U-shaped tapered no-core fiber (UTNCF) device, as illustrated in Figure 2c, is developed.^37^ Further bending the NCF into a U-shape enhances the evanescent field overlap with the surrounding medium, leading to improved RI sensitivity. Another variation of the microfiber interferometer is the microfiber knot resonator (MKR), as depicted in Figure 2d.^38,39^ Compared with interferometric devices, the runway-type MKR exhibits enhanced light–matter interaction efficiency owing to its ring-shaped configuration. Another example is the optical microfiber coupler (OMC) configuration.^40,41^ Unlike a conventional microfiber interferometer, an OMC consists of two or more microfibers that are precisely brought into close proximity, as illustrated in Figure 2e. When probe light is launched into the tapered region from the input port, the evanescent field enables optical coupling between adjacent microfibers, redistributing the optical power. According to the supermode theory,^42^ the parallel microfibers form a new composite waveguide that supports even and odd supermodes. When a single-polarized light is injected, both supermodes are excited simultaneously, accumulate a phase difference along the tapered region, and produce an interference spectrum at the output. The effective RI difference (Δ*n*_eff_) between the even and odd supermodes determines the interference pattern. Importantly, the effective group index difference between the even and odd supermodes for TE/TM polarization can be expressed as

4 $$G=\Delta n_{eff}-\lambda_{k}\frac{\partial \left(\Delta n_{eff}\right)}{\partial \lambda }$$

where λ_k_ is the wavelength of the *k*-th interference dip. By adjusting the microfiber diameter, parameter *G* can be tuned from positive to negative, passing through a dispersion turning point (DTP) where the sensor theoretically achieves infinite sensitivity to surrounding RI changes. Using a hybrid fusion–elongation–wet etching technique, an OMC with a taper waist diameter of 2.46 μm was fabricated, exhibiting a DTP near 1300 nm.^41^ The bare OMC achieved a high RI sensitivity of 9.1 × 10^4^ nm/RIU, which was further enhanced to −1.7 × 10^5^ nm/RIU after MXene modification.

### Mach–Zehnder and Modal Interferometric Sensors

Beyond microfiber and coupler-based interferometers, another important class of optical fiber interferometers used in MXene-integrated sensing involves Mach–Zehnder and modal interferometers. In practice, distinguishing between the two has become increasingly difficult as advances in fiber microfabrication, such as fusion splicing, femtosecond laser micromachining, and the development of specialty fibers, have enabled compact in-line mode couplers and converters. These structures allow controlled excitation and recombination of core and cladding modes within a single fiber strand, effectively realizing Mach–Zehnder or modal interference without the need for discrete optical paths. The resulting architecture combines simple fabrication with high sensitivity, providing versatile platforms for integrating functional materials such as MXenes for chemical and biosensing applications. Several representative configurations have been developed based on this principle. As shown in Figure 2f, one example is an MZI based on a lateral-offset configuration.^43^ The device consists of a lead-in SMF, a lead-in NCF, a thin NCF section, a lead-out NCF, and a lead-out SMF. Light propagating through the thin NCF acts as the reference path, while light transmitted across the air cavity formed by the lateral offset serves as the sensing path, thereby forming an interferometer. The air cavity can accommodate aqueous analytes, providing a confined region for efficient light–matter interaction. The use of the NCF as a mode exciter ensures strong optical field excitation, enabling the device to exhibit high RI sensitivity to changes in the surrounding environment. A slight variation of this structure is to replace the thin NCF with a thin core fiber (TCF).^44^

To improve the mechanical robustness of the structure, a modified configuration has been reported, as shown in Figure 2g.^45^ This device employs optical fibers with identical outer diameters (125 μm), thereby enhancing its mechanical strength. To increase sensitivity, the central TCF is intentionally offset by approximately 20 μm to excite stronger cladding modes and, consequently, a more pronounced evanescent field. The offset splice simultaneously forms a small air cavity that provides a confined space for the analyte to interact with the guided light. Another example of a modal interferometer is illustrated in Figure 2h, based on the well-known SNMNS (single-mode–no-core–multimode–no-core–single-mode) configuration. Instead of being used for RI sensing, this structure, with and without an MXene coating, was characterized for temperature measurement.^46,47^ Replacing the central multimode fiber (MMF) with an SMF yields the SMSMS configuration, as shown in Figure 2i.^48^ A simpler design based on a balloon-shaped modal interferometer has also been adopted for MXene sensor development (Figure 2j).^49^ The fabrication of this device is straightforward, involving only the bending of a standard SMF into a balloon shape. When light propagates through the bent region, a portion couples into the cladding, and interference between the cladding and core modes produces the output interference pattern. Variations in the surrounding refractive index alter the effective RI of the cladding modes, leading to spectral shifts. Further polishing of the bent section using a CO_2_ laser to remove part of the cladding enhances the evanescent field and thus improves the device sensitivity. In addition to directly measuring the RI of the surrounding medium, the balloon-shaped device was coated with a layer of polydimethylsiloxane (PDMS), whose RI exhibits strong temperature dependence due to its large thermo-optic coefficient. This modification transforms the device into a highly sensitive photothermal sensor, capable of detecting specific molecules that exhibit pronounced photothermal effects.

### Fabry–Perot Interferometric Sensors

FPI sensors represent a versatile and powerful class of optical devices that have been widely employed for the detection of physical, chemical, and biological parameters.^50^ An FPI typically consists of two parallel reflecting surfaces that form an optical cavity, where multiple reflections of light between these surfaces generate interference fringes. In this configuration, the light reflected from the first surface acts as the reference path, whereas the light reflected from the second surface serves as the sensing path. Variations in the cavity length or RI of the cavity medium alter the phase difference between the two reflected beams, leading to measurable spectral shifts or intensity variations in the interference pattern. Compared with other types of interferometers, FPIs offer the advantages of a compact structure, flexible design, and straightforward integration into fiber-based sensing systems. However, because of the strong optical absorption of MXenes in the visible and near-infrared regions, it is challenging to employ MXenes directly as the cavity medium for constructing a highly sensitive FPI. To overcome this limitation, a composite cavity structure was developed by combining MXenes with PDMS, which was subsequently attached to the cleaved end of an SMF to form an FPI sensor, as illustrated in Figure 2k. By leveraging the large thermo-optic coefficient of PDMS and the high thermal conductivity of MXenes, this composite structure enables the realization of a high-sensitivity and fast-response temperature sensor.^51^

### MXene Integration

The integration of MXene layers onto the cylindrical surface of optical fibers is a crucial step in achieving high-performance MXene-based fiber sensors. The deposition process directly influences the optical coupling efficiency, adhesion, and stability of the MXene coating, which in turn affects the overall sensitivity and repeatability of the sensor. The fabrication strategies employed for MXene-based interferometric fiber sensors can be broadly categorized into three main approaches: optical deposition, surface modification, and drop casting methods. Optical deposition exploits the optical field in the fiber in the form of an evanescent field to attract and immobilize MXene flakes onto the sensing region. Typically, a high-power light source (e.g., >30 mW) is connected to the as-fabricated interferometer device, where the sensing region is surrounded by MXene flakes. Because of the strong evanescent field that interacts with surrounding MXene, light-induced photothermal and optical gradient forces facilitated the adhesion of nanosheets along the sensing region. For instance, Bi et al. demonstrated the optical deposition of Nb_2_CT_*x*_ nanosheets onto a microfiber interferometer, specifically along the tapered region of an SMF.^52^ Similarly, Yi et al. employed optical deposition to integrate Ti_3_CNT_*x*_ MXene onto a TNCF within a lateral-offset-based MZI,^43^ as shown in Figure 3a. The bare sensor exhibits a smooth surface, whereas the surface roughness significantly increases after MXene incorporation; darkened regions observed at the offset junction further confirm the successful deposition of MXene flakes in the sensing region. Another representative example, shown in Figure 3b, involves the coating of V_2_CT_*x*_ MXene on the surface of an MKR via optical deposition.^39^

**Figure 3:**
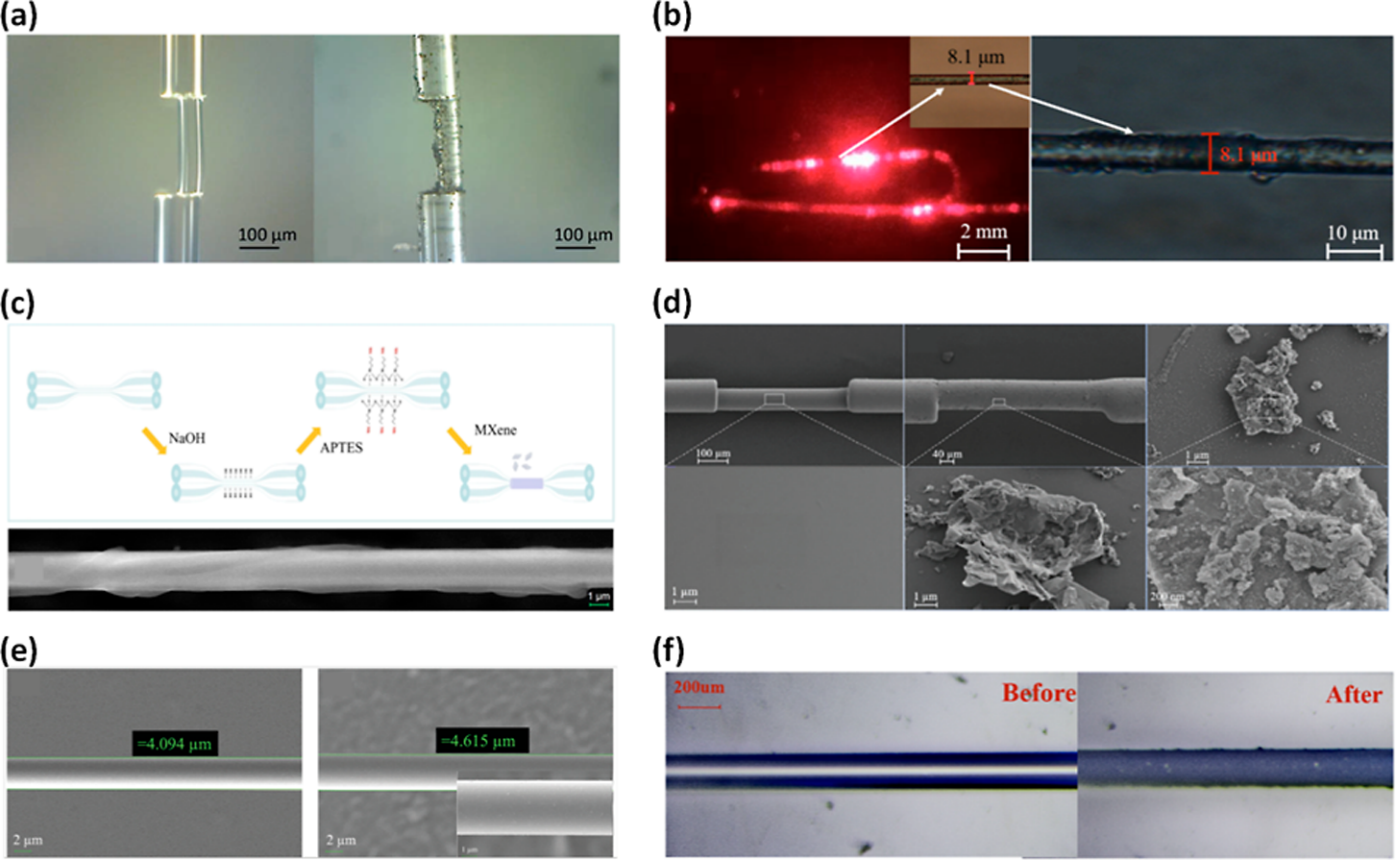
Deposition of MXenes on interferometric fiber-optic devices. (a) Ti_3_CNT_*x*_ MXene deposited on a TNCF-based MZI via optical deposition. Adapted with permission from ref 43. Copyright 2021 Optica Publishing Group. (b) V_2_CT_*x*_ MXene deposited on a microfiber-based MKR via optical deposition. Reproduced from ref 39. Available under a CC-BY XX license. Copyright 2023 Qing Wu. (c) Schematic illustration of the surface-modification process for depositing Ti_3_C_2_T_*x*_ MXene on an OMC and corresponding SEM characterization. Adapted with permission from ref 41. Copyright 2024 Optica Publishing Group. (d) Surface morphology of the MZI sensor before and after Ti_3_C_2_T_*x*_ MXene coating via the surface-functionalization technique and morphology of the fiber after the addition of gold nanoparticles (from left to right). Adapted with permission from ref 44. Copyright 2024 John Wiley & Sons. (e) Microscopy images of the microfiber surface before and after Ti_3_C_2_T_*x*_ MXene deposition by drop-casting. Adapted with permission from ref 53. Copyright 2025 Taylor & Francis. (f) Microscopy images of the fiber surface before and after V_2_CT_*x*_ MXene deposition by drop-casting. Adapted with permission from ref 47. Copyright 2025 Elsevier.

Surface functionalization techniques are used to enhance the binding affinity between the fiber surface and MXene nanosheets. The fiber surface can be pretreated with silane-based coupling agents to introduce hydroxyl or amino groups, promoting chemical bonding with MXene terminations. Peng et al. demonstrated surface modification of an OMC using chemical bonding and physical adsorption to immobilize Ti_3_C_2_T_*x*_ MXene, achieving a stable coating on the tapered region, as shown in Figure 3c.^41^ Scanning electron microscopy (SEM) revealed a distinct boundary between the light-colored, wrinkled MXene coating and the underlying OMC, confirming the successful bonding of MXene flakes to the fiber surface. Jin et al. further reported the deposition of Ti_3_C_2_T_*x*_ MXene nanosheets onto a TCF-based MZI via surface functionalization.^44^ As illustrated in Figure 3d, comparison between the microscopic images of the uncoated and MXene-coated sensors verifies the formation of a MXene coating on the fiber surface. Moreover, the MXene film serves as an excellent functional platform for anchoring gold nanoparticles, enabling the formation of MXene@Square Gold Nanoparticles (SGNps) composites that further enhance light–matter interaction and improve sensing sensitivity.

Drop-casting represents the simplest and most straightforward method for depositing MXenes onto optical fibers. Mustaffa et al. reported the fabrication of Ti_3_C_2_T_*x*_ coatings on tapered fibers using a controlled drop-casting process, as illustrated in Figure 3e.^53^ In another example, Zhou et al. employed a drop-casting technique to deposit V_2_CT_*x*_ MXene onto an MZI, as shown in Figure 3f.^47^ In their approach, V_2_CT_*x*_ powder was first dispersed in a LiCl solution, and the resulting V_2_CT_*x*_–LiCl mixture was applied to the sensing region and subsequently dried, forming an adherent MXene-based sensitive layer on the fiber surface.

Table 1 summarizes the comparison among the three MXene deposition approaches, highlighting their fabrication principles, advantages, and limitations. Among these approaches, surface modification generally provides the most uniform and stable MXene coating because of the strong chemical bonding between surface-functionalized silica and MXene terminations. This method ensures a compact film morphology with minimal scattering loss and excellent long-term adhesion. In contrast, optical deposition enables spatially selective coating on tapered or microstructured regions by exploiting the evanescent optical field, but it often results in nonuniform or discontinuous layers due to uneven flake aggregation driven by the optical-field intensity. Meanwhile, drop-casting remains the simplest and most scalable approach, capable of forming continuous coatings over large areas. However, their reproducibility and film compactness are highly dependent on parameters such as MXene concentration, dispersion viscosity, and drying conditions. In addition, the interfacial bonding between MXenes and the silica surface has not yet been fully elucidated, and further investigation is required to assess the long-term stability and reliability of such coatings. Overall, the selection of the deposition method involves a trade-off among film uniformity, coating precision, and fabrication simplicity, which should be optimized according to the sensor configuration and target application.

**Table 1:** Summary and Comparison of MXene Deposition Methods for Interferometric Optical Fiber Sensors

Deposition Method	Principle/Process	Advantages	Limitations
Optical deposition	Light propagating within the fiber generates localized photothermal and gradient forces that attract MXene flakes to the evanescent field region for adhesion	Enables selective coating at specific fiber locations; nondestructive to the fiber structure	Poor uniformity and reproducibility; film thickness difficult to control
Surface modification	Chemical treatment introduces reactive groups on the silica surface to bond with MXene terminations via covalent interactions	Excellent film uniformity and adhesion; low scattering loss; high repeatability	Requires multiple surface treatment steps; higher fabrication complexity
Drop-casting	Fiber is immersed or coated with MXene dispersion, followed by drying to form thin or multilayer coatings; thickness controlled by solution concentration and withdrawal speed	Simple, low-cost, and scalable; compatible with various fiber geometries	Film aggregation or cracking during drying; weaker adhesion compared to chemical methods

### Sensitivity Enhancement

The incorporation of MXene layers into optical fiber interferometers not only extends sensing functionalities but also significantly enhances the overall sensitivity. This improvement arises from the unique physicochemical characteristics of MXenes, including high electrical conductivity, large specific surface area, tunable RI, and a rich surface chemistry. When integrated onto the sensing region of an interferometer, MXene modifies the local electromagnetic field distribution, strengthens evanescent-field coupling, and provides abundant active sites for adsorption. These interactions amplify the optical response to external stimuli and translate minute physical or chemical perturbations into measurable spectral shifts or intensity variations.

In humidity sensing, the hydrophilic and layered nature of MXenes plays a dominant role. The abundant surface terminations and interlayer spacing in MXenes serve as active adsorption sites for water molecules. Upon exposure to humid air, physisorption and chemical bonding between water molecules and surface functional groups induce interlayer expansion and formation of a transient hydrogen-bond network, resulting in variations of both the effective RI and conductivity of the MXene coating. In interferometric configurations such as tapered SMF or NCF, these changes modulate the effective RI difference and produce distinct spectral shifts. At low relative humidity (RH), water molecules are rapidly adsorbed, decreasing the effective RI of the MXene layer; at higher humidity, swelling and structural rearrangement reverse the trend, yielding nonlinear but highly sensitive responses.^36,52^ Monotonic responses were also observed across a wide RH range, with saturation occurring beyond 80% RH where no further response was detected.^37^ These findings confirm that the intrinsic hydrophilicity and humidity-responsive microstructure of MXenes effectively amplify light–matter coupling, offering a promising pathway toward the development of fast-response, high-sensitivity humidity sensors based on interferometric configurations with strong evanescent fields.

For RI and related biosensing applications, MXene coatings enhance sensitivity by increasing evanescent-field penetration and providing abundant molecular adsorption sites. The metallic conductivity and broadband optical absorption of MXenes intensify the local electromagnetic field at the fiber–coating interface, allowing even small RI or adsorption-induced perturbations to produce pronounced spectral changes. As illustrated in Figure 4a, a MXene-based salinity sensor provides a representative example.^43^ Without MXenes, the evanescent field in the sensing region interacts only weakly with NaCl molecules at the fiber surface. After MXene deposition, the layered and conductive coating allows the evanescent field to penetrate through the MXene film with increased depth, while the large specific surface area and abundant functional groups enable efficient capture of ions near the interface. These two factors jointly enhance the light–matter interaction, resulting in a larger change in the effective RI difference within the interferometer and consequently stronger spectral variation. The sensitivity enhancement factor was found to depend strongly on the MXene concentration, with higher concentrations yielding greater sensitivity, as shown in Figure 4b. However, excessive MXene loading also increases the coating thickness, which in turn leads to larger insertion loss caused by enhanced optical scattering and absorption (Figure 4c). Further improvement has been realized by integrating gold nanoparticles with MXene layers to form composite coatings.^44,48^ The MXene surface provides nucleation sites and strong anchoring for gold nanoparticles, expanding effective binding sites on the sensor surface. Meanwhile, the localized surface plasmon resonance (LSPR) triggered by the gold nanoparticles interacts with the broadband optical modes of MXenes, producing strong local-field enhancement and amplified charge-transfer dynamics, thus further improving sensitivity. Additionally, MXenes also support surface plasmon polaritons (SPPs), electromagnetic waves confined at the MXene–dielectric interface, providing a new mechanism for sensitivity enhancement in microfiber interferometers.^27,54^ Their high carrier density, metallic conductivity, and tunable dielectric constant enable plasmonic excitation across the near- and mid-infrared ranges. When coated onto optical microfibers, MXene films such as Ti_3_C_2_T_*x*_ sustain SPP propagation and strong field localization, significantly improving sensitivity to surrounding gases or liquids. In one example, a Ti_3_C_2_T_*x*_-coated microfiber sensor exhibited wavelength red shifts proportional to ammonia gas concentration, achieving a detection limit of 100 ppm and a response time below 100 s. Theoretical analysis showed that thicker MXene coatings enhance the SPP quality factor and extend propagation length, while density functional theory calculations revealed strong hydrogen bonding between NH_3_ and oxygen-terminated MXene surfaces, explaining the high selectivity toward ammonia. The results indicate that MXene-supported SPPs could enable metal-free plasmonic fiber sensors operating in the 1.55 μm band with high selectivity and compatibility with standard optical-fiber platforms.

**Figure 4:**
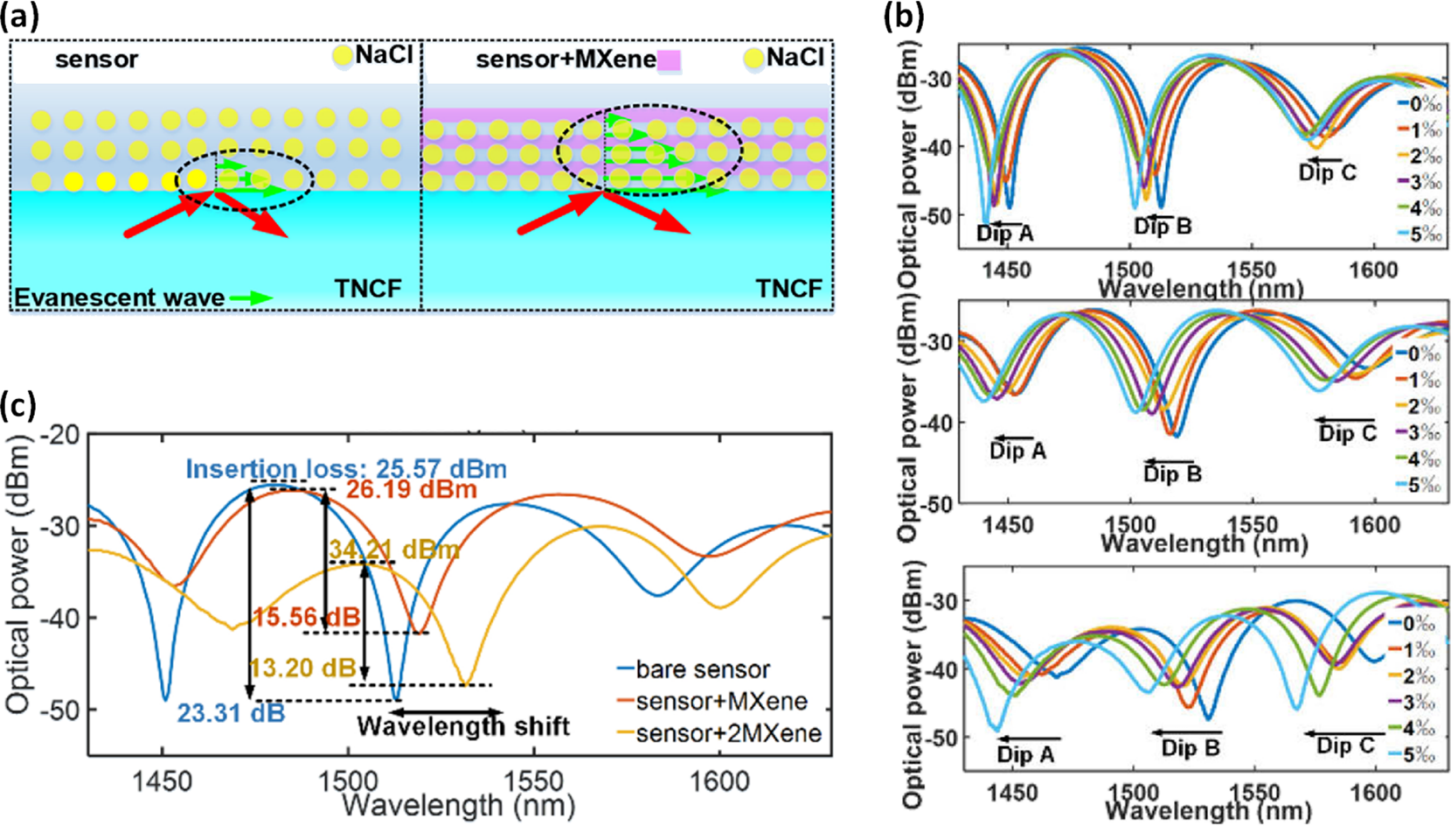
MXene-enabled sensitivity enhancement of an optical fiber salinity sensor. (a) Schematic illustration of the sensitivity enhancement mechanism. TNCF: thin no-core fiber. (b) Transmission spectra of the bare sensor, the sensor coated with MXenes (0.5 mg/mL), and the sensor coated with a higher MXene concentration (1 mg/mL), from top to bottom, under varying salinity conditions. (c) Initial transmission spectra of the sensor with 0.5 mg/mL MXene (denoted as MXene) and 1 mg/mL MXene (denoted as 2MXene). Adapted with permission from ref 43. Copyright 2021 Optica Publishing Group.

In temperature sensing, MXenes enhance response speed and sensitivity through their high thermal conductivity and temperature-dependent optical constants, respectively. Owing to the strong metallic bonding and efficient phonon transport, MXenes (e.g., Ti_3_C_2_T_*x*_ and V_2_CT_*x*_) exhibit thermal conductivities several times greater than that of silica, allowing rapid heat transfer across the sensing region. When coated on microfibers or incorporated into Fabry–Perot or Mach–Zehnder configurations, MXenes ensure uniform heat distribution and faster thermal equilibrium.^47,51^ Their temperature-dependent RI and thermal expansion further modulate the effective optical path length, improving wavelength or intensity sensitivity.^38,46^ These demonstrations indicate that MXenes serve as both efficient thermal conduits and thermo-optic modulators, enabling fast-response and highly sensitive temperature measurements.

Table 2 summarizes the specific enhancement factors achieved through the MXene integration in interferometric fiber sensors. Across these different sensing modalities, MXenes enhance the performance through their intrinsic material properties and interfacial interactions. Hydrophilicity and structural modulation dominate humidity sensing; molecular adsorption and plasmonic coupling amplify RI detection; high thermal conductivity and thermo-optic effects improve temperature response; and SPP excitation enables strong field confinement for gas sensing. Together, these mechanisms establish MXenes as transformative material platforms for high-sensitivity, multifunctional optical fiber interferometric sensors. These enhancement mechanisms similarly govern the performance improvements observed in other MXene-integrated fiber sensor configurations, as detailed below.

**Table 2:** Sensitivity Enhancement of MXene-Coated Interferometric Optical Fiber Sensors

Sensing Type	Sensor Configuration	MXene Type and Integration	Enhancement Factor (%)	Ref
Humidity	Microfiber interferometer	Ti_3_C_2_T_*x*_ – drop casting	187	^53^
RI/Hemoglobin	Microfiber interferometer	Nb_2_CT_*x*_ – optical deposition	7	^35^
RI/Oxacillin sodium	Microfiber interferometer	Ti_3_CNT_*x*_ + PDMS – optical deposition	5	^55^
RI/Streptomycin sulfate	OMC	Nb_2_CT_*x*_ – optical deposition	55	^40^
RI/Salinity	TNCF-MZI	Ti_3_CNT_*x*_ – optical deposition	137	^43^
RI/Riboflavin	TCF-MZI	Ti_3_C_2_T_*x*_ + gold nanoparticles – surface modification	79	^44^
RI/Cytochrome C	TCF-MZI	Ti_2_CT_*x*_ – surface modification	54	^45^
RI/DNA	SMF-MZI	Ti_3_C_2_T_*x*_ + gold nanoparticles – surface modification	69	^48^
RI/Antibiotics	Balloon-shaped interferometer	MXene + PDMS – optical deposition	281	^49^
RI	OMC	Ti_3_C_2_T_*x*_ – surface modification	87	^41^
Temperature	MMF-MZI	Ti_3_C_2_T_*x*_ – surface modification	57	^46^
Temperature	MKR	V_2_CT_*x*_ – optical deposition	1500	^38^
Temperature	MMF-MZI	V_2_CT_*x*_ – drop casting	43	^47^

## Fiber Grating-Based Sensing

Optical fiber gratings represent an important class of in-fiber photonic structures that have been extensively utilized for physical, chemical, and biological sensing owing to their compact geometry, wavelength-encoded response, and compatibility with standard single-mode fibers.^56–58^ Their sensing principle relies on resonant-mode coupling induced by the periodic RI modulation along the fiber core. Variations in the surrounding environment modify the effective refractive indices of the guided modes, leading to measurable wavelength or intensity shifts. However, a conventional fiber Bragg grating (FBG) is inherently insensitive to the ambient medium, such as RI changes, because the guided light remains well-confined within the fiber core. To enable a strong interaction between the guided modes and the external medium, cladding-mode excitation is required. Two configurations, namely, the long-period grating (LPG)^59^ and the tilted fiber Bragg grating (TFBG),^60^ have been developed for this purpose. Because of their surface-accessible cladding modes and strong evanescent fields, both LPGs and TFBGs provide excellent platforms for MXene integration and have been successfully employed in the development of MXene-based fiber sensors.

### Long-Period Gratings

An LPG consists of a periodic modulation of the core RI of an SMF with a pitch of several hundred micrometers and an overall length of a few centimeters, as shown in Figure 5a. The periodic index modulation couples the propagating fundamental core mode to a set of copropagating cladding modes, producing a series of attenuation bands at discrete wavelengths, known as resonant wavelengths, in the transmission spectrum. Each attenuation band corresponds to coupling between the core mode and a specific cladding mode, each exhibiting a distinct sensitivity to environmental perturbations. Generally, longer resonant wavelengths and higher-order cladding modes yield higher RI sensitivity. The *m*-th order resonant wavelength satisfies the phase-matching condition

5 $$\lambda_{res,m}=\left(n_{core}-n_{cladding,m}\right)\Lambda$$

where *n*_core_ and *n*_claddingg,m_ are the effective refractive indices of the core and LP_0m_ cladding modes, respectively, and Λ denotes the grating period. When the surrounding RI changes, the RI of the cladding modes is altered, resulting in a corresponding shift of the resonant wavelength. When a MXene film is coated onto the grating region, its high RI and strong optical absorption enhance the evanescent-field interaction between the cladding modes and the surrounding medium. The layered structure and rich surface functionality of MXene provide abundant adsorption sites, enabling charge transfer and molecular binding that amplify the spectral response. Using optical deposition, a MXene layer can be uniformly deposited onto the surface of an LPG, as illustrated in Figure 5b. When combined with functional polymers, the resulting polymer-functionalized MXene coatings offer additional selectivity and improved sensitivity for specific analytes. Additionally, a distinctive feature of LPGs is the existence of a turn-around point (TAP), also referred to as the turning point, in the phase-matching condition for high-order cladding modes, where the sensor exhibits its maximum RI sensitivity.^61,62^ The TAP occurs when two resonant wavelengths corresponding to the same cladding mode coincide, forming a U-shaped attenuation band in the transmission spectrum. This condition can be realized by precisely selecting the grating pitch during fabrication. The transmission power and bandwidth of the U-shaped attenuation band are highly dependent on the surrounding medium, enabling ultrahigh RI sensitivity with resolutions approaching the parts-*per*-million level. When the surrounding RI increases significantly, the LPG response may deviate from the TAP, causing the single attenuation band to split into two distinct resonant dips. The separation between these dips can thus serve as an additional indicator for RI sensing. Owing to the extremely sharp dispersion and strong mode coupling near the TAP, such TAP-LPGs provide an excellent platform for MXene integration, offering opportunities for the development of ultrasensitive chemical and biological sensors.

**Figure 5:**
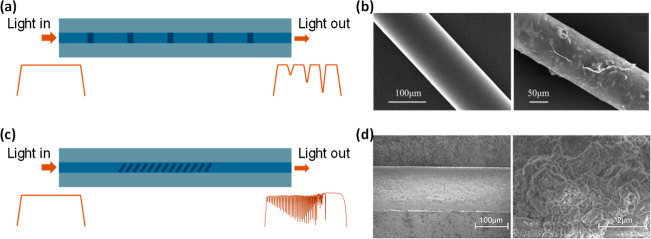
Grating–MXene fiber sensor. (a) Schematic of an LPG. (b) SEM images of a bare LPG and a Ti_3_C_2_T_*x*_-coated LPG (from left to right). Adapted with permission from ref 63. Copyright 2023 Elsevier. (c) Schematic of a TFBG. (d) SEM image of the lateral surface of a TFBG coated with a Ti_2_C/C_60_ composite film and zoomed-in SEM image of the Ti_2_C/C_60_ composite film (from left to right). Adapted with permission from ref 64. Copyright 2024 Optica Publishing Group.

### Tilted Fiber Bragg Gratings

In contrast to LPGs, TFBGs couple the forward-propagating core mode to backward-propagating cladding modes through a small tilt of the grating planes with respect to the fiber axis. This tilt breaks the axial symmetry of the structure, producing a dense comb of cladding-mode resonances that are highly sensitive to variations in the surrounding RI, as illustrated in Figure 5c. The evanescent fields of these cladding modes extend into the surrounding medium, making TFBGs ideal for ambient RI sensing by monitoring spectral shifts or intensity changes of the cladding-mode resonances. The integration of MXenes further enhances TFBG performance by exploiting their metallic conductivity, broadband optical absorption, and tunable surface terminations, which collectively increase light–matter interaction and surface reactivity. Figure 5d shows SEM images of a Ti_2_CT_*x*_/C_60_ composite film coated onto a TFBG, developed for humidity sensing. The nanoporous structure of the Ti_2_CT_*x*_/C_60_ composite provides a large surface area and facilitates rapid vapor diffusion, thereby enhancing the interaction between the evanescent field and ambient humidity and improving the overall sensitivity and response speed of the sensor. Another important feature of TFBGs is their capability for multiparameter sensing, such as the simultaneous measurement of RI and temperature. This is enabled by the distinct sensitivities of the core and cladding modes: the core mode primarily responds to temperature changes while remaining insensitive to ambient RI variations, whereas the cladding modes are sensitive to both parameters. By using the core-mode resonance as an internal reference, it becomes feasible to decouple these responses and achieve accurate multiparameter sensing.^65^

### Evanescent Field-Based Sensing

Light propagates through an optical fiber primarily via total internal reflection, which confines the optical field within the fiber core and enables long-distance transmission with minimal loss. However, for sensing applications, it is often desirable to intentionally allow a fraction of the guided light to leak out of the core in the form of an evanescent field that extends into the surrounding medium. This evanescent field facilitates direct light–matter interaction, allowing variations in the RI, absorption, or chemical composition near the fiber surface to modulate the transmitted or reflected signal.^66^ To achieve strong field exposure, various fiber geometries, such as tapered fibers, D-shaped fibers, U-shaped fibers, and side-polished fibers, are employed to enhance the overlap between the guided mode and the external environment. These configurations provide large surface areas and strong optical field penetration, making them highly effective for detecting subtle environmental changes and particularly suitable for surface functionalization and integration with advanced 2D materials. MXenes are highly effective coating materials owing to their high RI, metallic conductivity, and abundant surface terminations, which together promote efficient light–matter interaction and analyte adsorption within the evanescent-field region. In this section, we review several evanescent-field-based sensing configurations that have been integrated with MXene materials to achieve enhanced sensing performance.

One of the simplest approaches to enhancing the evanescent field of an optical fiber is to remove part or all of the cladding, creating a cladding-reduced section where the optical field extends into the surrounding environment. This configuration enables direct light–matter interaction between the guided mode and external analytes. A commonly used fabrication technique is chemical wet etching, in which the optical fiber is immersed in a chemical solution, typically hydrofluoric acid, ammonium fluoride, or their mixtures, to selectively remove cladding material at a controlled rate. As illustrated in Figure 6a, the resulting etched SMF can serve as an efficient evanescent-field sensor. By adjusting the immersion time, the remaining fiber diameter can be precisely controlled, thereby tuning the strength of the evanescent field and the overall sensitivity. A simple transmission-based interrogation system can be constructed using a light source and a power meter, providing a low-cost and compact sensing platform. Through the deposition of MXene layers such as V_4_C_3_T_*x*_ and Ti_3_C_2_T_*x*_ onto the etched region, high-sensitivity humidity sensors have been demonstrated.^67,68^ The enhancement mechanism originates from MXene’s intrinsic hydrophilicity and large specific surface area. When exposed to humid air, water molecules are adsorbed within the interlayer spacing of the MXene, altering its electric susceptibility and modifying the evanescent-field distribution. This manifests as measurable changes in transmission intensity. In addition, water adsorption enhances charge-transfer interactions, which modify the imaginary component of MXene’s RI and increase optical absorption, leading to a further decrease in transmitted optical power.

**Figure 6:**
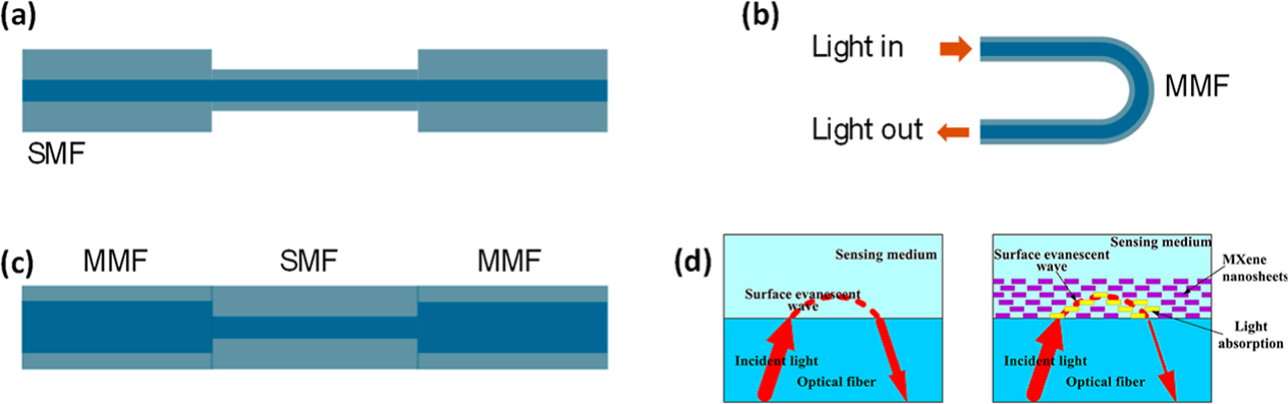
Evanescent field-based optical fiber sensors. (a) Etched single-mode fiber (SMF). (b) U-shaped multimode fiber (MMF). (c) Heterostructure formed by an MMF–SMF–MMF configuration. (d) Comparison of light absorption of surface evanescent waves for sensors without (left) and with (right) the MXene layer. Adapted with permission from ref 72. Copyright 2020 American Chemical Society.

Another effective approach to strengthening the evanescent field is to introduce a U-shaped (or U-bent) configuration (Figure 6b), where the optical fiber is bent to a small radius such that part of the guided light penetrates through the cladding into the surrounding medium. The bending induces mode deformation and radiation loss, significantly enhancing the evanescent field intensity and extending its reach beyond the cladding boundary. The strength of the evanescent field depends strongly on the bend radius; smaller radii typically produce greater field penetration and therefore higher sensitivity. When the surrounding RI changes, the boundary conditions at the interface are altered, resulting in measurable variations in transmission intensity.^69^ To facilitate efficient coupling between the guided mode and the surrounding medium, MMFs with reduced cladding thickness, for instance, fibers with 200/220 μm core/cladding diameters, are often employed.^69^ By coating the U-shaped region with an MXene-based film, the strong field localized near the fiber surface interacts directly with the high-index, broadband-absorbing MXene layer. The layered structure and surface terminations of MXenes facilitate adsorption and charge transfer with target molecules, while their metallic conductivity and tunable dielectric constant enhance the optical field confinement at the interface. Incorporating secondary materials such as TiO_2_ nanoparticles or polymers further improves sensing performance by increasing surface area, porosity, and chemical selectivity.^69–71^

Another effective configuration to enhance the evanescent field interaction is the core–core mismatch structure, typically realized by splicing a short section of an SMF between two MMFs, as shown in Figure 6c. This heterostructure arrangement causes a portion of the guided light from the MMF to couple into the cladding of the SMF due to mode-field mismatch, creating strong surface evanescent waves along the SMF section. The resulting cladding modes are highly sensitive to RI variations in the surrounding environment. By monitoring changes in the transmitted intensity or spectral profile, these environmental variations can be accurately quantified. Since this design does not require etching and offers high repeatability, it preserves mechanical robustness while maintaining excellent optical accessibility. Thus, mismatch-based heterostructure fibers represent a simple yet powerful route for implementing optical fiber sensors with broad applicability in biochemical sensing. When an MXene layer is deposited onto the exposed SMF surface, its metallic conductivity and wideband optical absorption enhance the penetration and scattering of the evanescent field. The abundant surface terminations and multilayered MXene structure significantly enhance analyte absorption and allow analyte penetration between layers, facilitating efficient light–matter coupling, further enhancing measurement sensitivity,^72^ as illustrated in Figure 6d.

## Surface Plasmon Resonance-Based Sensing

### Surface Plasmon Resonance

Evanescent-field optical fiber probes provide compact and portable alternatives to conventional prism-based SPR systems. By confining the optical excitation and plasmonic interaction within the micro- or nanoscale dimensions of an optical fiber, these probes enable in situ, real-time detection with high sensitivity and spatial precision. In a typical configuration, an SPR-based optical fiber sensor is realized by coating a thin layer of a noble metal, such as gold, onto the polished, tapered, or unclad region of the fiber. When light propagates through the fiber, the evanescent field penetrates the glass–metal interface, where it can couple to collective charge oscillations at the metal surface, known as SPPs. Resonant coupling occurs when the propagation constants of the evanescent wave and the SPPs are matched, resulting in a distinct attenuation (or dip) in the transmitted spectrum, referred to as the plasmon resonance condition. The resonance wavelength is highly sensitive to the RI of the dielectric medium adjacent to the metal film, allowing small environmental perturbations, such as molecular adsorption or biochemical binding events, to be detected through spectral shifts. Compared with traditional prism-based Kretschmann configurations, fiber-based SPR sensors offer several advantages, including compactness, mechanical flexibility, remote sensing capability, and straightforward integration with microfluidic or biochemical interfaces. Depositing layers of MXene onto the metal film can further enhance the sensitivity of optical fiber SPR sensors by simultaneously improving plasmonic coupling efficiency and surface interaction with analytes. The high electrical conductivity of MXenes enables strong electromagnetic coupling at the metal–dielectric interface, thereby facilitating more efficient excitation of SPPs. In addition, MXenes possess high RI and broadband optical absorption, modifying the local optical field distribution near the metal surface and amplifying the field intensity within the sensing region. These effects increase the overlap between the plasmonic field and the surrounding medium, resulting in stronger resonance shifts in response to RI variations. The surface chemistry of MXenes also plays a crucial role in performance enhancement. The abundant surface terminations and large specific surface area provide numerous active sites for molecular adsorption or chemical binding, improving both sensitivity and selectivity toward specific analytes.

Figure 7 summarizes the evanescent-field fiber structures that have been employed in the development of MXene-enhanced SPR sensors. As shown in Figure 7a, a plastic-clad MMF can be used as a simple SPR platform by removing its polymer coating to expose the evanescent field for metal or MXene deposition. The TFBG configuration, illustrated in Figure 7b, also supports SPR excitation due to its strong cladding modes, which provide efficient coupling to plasmonic layers. Another widely adopted geometry is the D-shaped (or arc-shaped) fiber, obtained by side-polishing an SMF to partially or completely remove the cladding, as shown in Figure 7c. The planar region formed by polishing allows uniform deposition of metal films and MXene layers, ensuring strong light–matter interaction at the exposed surface. In addition to these geometries, heterostructure fiber configurations are frequently employed in SPR sensor design. By splicing a segment of a different type of fiber between two MMFs—such as an SMF (Figure 7d), a photonic crystal fiber (Figure 7e), or an NCF (Figure 7f)—mode-field mismatch occurs at the junctions, exciting cladding modes that generate strong evanescent fields. These fields can then be exploited to excite surface plasmons through thin-film metal deposition. Such heterocore structures offer flexibility in tailoring mode distribution and resonance strength while maintaining mechanical robustness. In addition to these conventional configurations, considerable efforts have also been devoted to numerical investigations of microstructured fiber devices for MXene-enhanced SPR sensing.^73–86^ It is interesting to note that although gold is typically used as the metal film for SPR excitation because of its high chemical stability, silver has also been employed in certain designs due to its lower cost than gold.^87^ In such cases, the subsequent MXene coating not only enhances the sensitivity but also serves as a protective layer that effectively prevents oxidation of the underlying silver film, thereby improving both performance and durability. It is worth noting that although previous studies have demonstrated that MXenes themselves can support SPPs in the C-band,^27,54^ in these configurations, the MXene film is primarily employed as an enhancement coating to improve the performance of conventional metal-film-based SPR sensors (e.g., gold or silver coatings).

**Figure 7:**
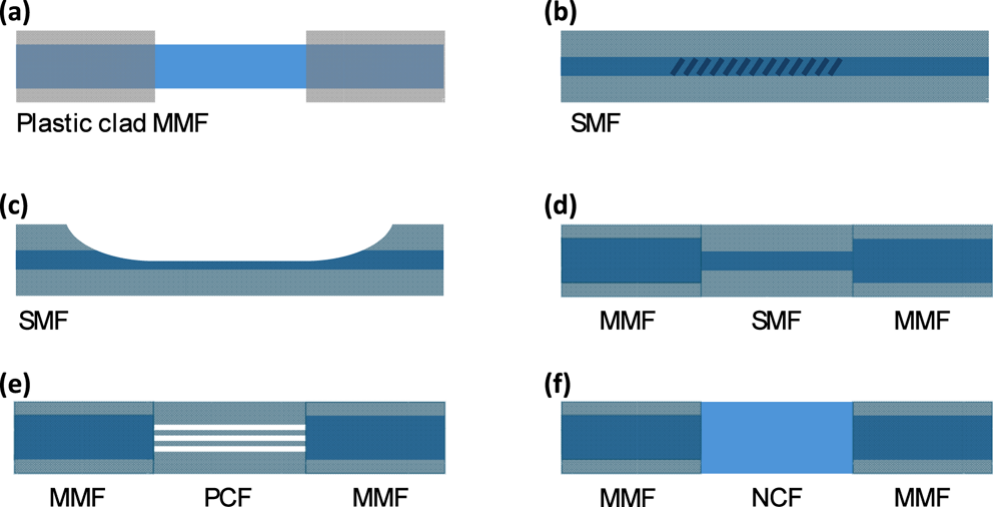
Schematic configurations of evanescent-field fiber structures employed for MXene-enhanced SPR sensing. (a) Plastic-clad multimode fiber (MMF) with the outer polymer coating removed to expose the evanescent field. (b) Tilted fiber Bragg grating (TFBG) supporting cladding-mode coupling for SPR excitation. (c) D-shaped (or arc-shaped) single-mode fiber (SMF) fabricated by side-polishing the cladding region. (d–f) Heterostructure configurations formed by inserting different fiber segments between two MMFs: (d) SMF, (e) photonic crystal fiber (PCF), and (f) no-core fiber (NCF).

In addition to sensitivity, another important performance metric for optical fiber SPR sensors is the figure of merit (FOM), defined as the ratio of the sensor’s sensitivity (S) to the full width at half-maximum (fwhm) of the resonance spectrum

6 $$FOM=\frac{S}{FWHM}$$

Typically, MXene-enhanced SPR sensors exhibit a higher FOM than their uncoated counterparts, primarily due to the increased sensitivity introduced by the MXene layer. As the thickness of the MXene coating increases, the sensitivity tends to improve owing to stronger plasmonic coupling and enhanced light–matter interaction. However, the fwhm of the resonance spectrum also broadens with increasing MXene thickness because of the higher optical losses associated with the coating.^88–93^ This trade-off can ultimately lead to a reduction in FOM at excessive coating thicknesses, thereby degrading the overall sensing performance. Therefore, the thickness of the MXene layer must be carefully optimized to balance the competing effects of sensitivity enhancement and spectral broadening, ensuring maximum performance improvement. More broadly, similar trade-offs between sensitivity enhancement and other sensing metrics, including insertion loss, spectral quality, stability, and response dynamics, also exist in other MXene-enhanced optical fiber sensing platforms, highlighting the importance of comprehensive device-level optimization rather than sensitivity enhancement alone.

### Localized Surface Plasmon Resonance

In contrast to propagating SPR, where lossy surface waves travel along a continuous glass–metal interface, LSPR arises from the collective oscillation of free electrons confined within metallic nanostructures such as nanoparticles, nanorods, or nanoholes. When illuminated by incident light, the conduction-band electrons in these nanostructures oscillate coherently with the electromagnetic field, giving rise to intense, spatially confined plasmonic fields. This phenomenon manifests as a characteristic extinction or scattering peak in the optical spectrum, whose wavelength and amplitude are highly sensitive to the RI of the surrounding medium and to the geometry, size, and material composition of the nanostructures. Compared with conventional SPR, LSPR offers several distinct advantages. The use of metallic nanostructures provides stronger field localization and a larger effective interaction area with the surrounding environment, thereby enhancing sensitivity to ambient RI variations. These features make LSPR particularly attractive for biochemical and environmental sensing, where molecular binding or adsorption at the nanoparticle surface leads to measurable spectral shifts. When integrated with optical fibers, LSPR-based configurations combine the miniaturization and remote accessibility of fiber platforms with the high near-field enhancement of metallic nanostructures. The evanescent field from the fiber core serves as an efficient excitation source for localized plasmonic modes on the fiber surface, enabling compact, flexible, and highly sensitive fiber-optic LSPR probes. The incorporation of MXene coatings or MXene–metal nanocomposites can further amplify the local field intensity, promote charge transfer, and increase analyte adsorption, leading to superior detection performance compared with conventional noble-metal nanoparticle coatings. Furthermore, the resonance condition of these hybrid plasmonic systems can be flexibly tuned across a wide spectral range, from the visible to the near-infrared, owing to MXene’s broadband optical absorption and wide plasmonic response window. These unique features make MXene-integrated LSPR fiber sensors, particularly those based on telecommunication fibers, highly versatile platforms for broadband, high-sensitivity chemical and biological detection.

To effectively excite LSPR within an optical fiber platform, the evanescent field must be sufficiently exposed to interact with metallic nanostructures. Therefore, standard SMFs are typically modified to enhance field penetration. Among various configurations, tapered fibers have been most widely employed for constructing MXene-enhanced LSPR sensors. Through surface modification, Ti_3_C_2_T_*x*_ MXene nanosheets can be uniformly deposited onto the microfiber surface, providing a conductive and chemically active layer.^94^ Subsequently, high-aspect-ratio gold nanorods are self-assembled onto the MXene coating via electrostatic interactions facilitated by the surface functional groups. The LSPR excited on the gold nanorods generates electron–hole pairs, which are efficiently transferred to the MXene layer as hot charge carriers, leading to intensified local electromagnetic fields at the fiber interface. This synergistic interaction significantly enhances the evanescent field strength and, consequently, the sensitivity of the sensor to minute RI variations at the MXene–metal–dielectric interface. In another configuration, a double S-tapered fiber coated with gold nanoparticles served as the LSPR excitation platform,^95^ where an additional Nb_2_CT_*x*_ MXene layer was subsequently deposited onto the gold nanoparticles to further enhance sensitivity. The hybrid structure exhibited superior field confinement and a pronounced increase in resonance depth compared with the bare metal-coated fiber. Beyond these conventional tapered structures, more sophisticated platforms, such as MMF–MCF–MCF–MMF composite fibers incorporating symmetric transverse offset splicing, etching, and waist-expanded tapering,^96^ as well as waveflex optical fiber geometries fabricated through sequential splicing and tapering,^97–100^ have been explored. These advanced architectures provide enhanced modal coupling and stronger field localization, enabling precise excitation of LSPR and improved sensing performance.

Table 3 summarizes the representative MXene-enhanced SPR and LSPR optical fiber sensors that have been reported to date. The high electrical conductivity, high optical absorption, and rich surface chemistry enable stronger plasmonic field confinement, improved charge-transfer efficiency, and increased surface adsorption, collectively leading to substantial sensitivity improvement. These results confirm MXene’s effectiveness as a multifunctional enhancement layer for plasmonic fiber sensors. It is also worth noting that by synergistically combining SPR, LSPR, and MXene-induced enhancement, for instance, through composite coatings that incorporate a gold film, an MXene layer, and gold nanoparticles/nanorods, the overall sensing performance can be further improved, enabling higher sensitivity.^92^

**Table 3:** Sensitivity Enhancement of MXene-Coated Optical Fiber SPR and LSPR Sensors

Sensing Type	Sensor Configuration	MXene Type and Integration	Enhancement Factor (%)	ref
RI	Heterostructure (SPR)	Ti_3_C_2_T_*x*_ – optical deposition	30	^72^
RIg differentiation factor 11	Plastic clad removed MMF (SPR)	Ti_3_C_2_T_*x*_ + gold nanoparticles – surface modification	164	^101^
RI/Saccharin and BHT	TFBG (SPR)	Ta_2_CT_*x*_ – optical deposition	23	^102^
RI	D-shaped fiber (SPR)	Ti_3_C_2_T_*x*_ – spin coating	54	^88^
RI/Melamine	TFBG (SPR)	Ta_2_CT_*x*_ – optical deposition	27	^103^
RI/Immunoglobulin G	Heterostructure (SPR)	Ti_3_C_2_T_*x*_ + gold nanoparticles – surface modification	32	^89^
RI/Temperature	Heterostructure (SPR)	Ti_3_C_2_T_*x*_ – drop coating	55	^87^
Lead ions	D-shaped fiber (SPR)	Ti_2_CT_*x*_ – surface modification	34	^104^
RI/Staphylococcus aureus	Heterostructure (SPR)	Ti_3_C_2_T_*x*_ – dip coating	61	^90^
RI/Alcohol	Heterostructure (SPR)	MXene + SnO_2_ – electrospinning	84	^105^
RI	D-shaped fiber (SPR)	Ti_2_CT_*x*_ – surface modification	128	^91^
RI/Acetylcholine	Plastic clad removed MMF (SPR)	Ti_3_C_2_T_*x*_ + gold nanorods – surface modification	218	^92^
RI/Renal cancer proteins and cells	Microfiber (LSPR)	Ti_3_C_2_T_*x*_ + gold nanorods – surface modification	68	^94^
Tyramine	Double S-tapered fiber (LSPR)	Nb_2_CT_*x*_ – surface modification	100	^95^

### Chemical and Biological Sensing Applications

Building upon the diverse sensing mechanisms discussed in the previous sections, MXene-functionalized optical fiber sensors have been extensively explored for a wide range of chemical and biological detection applications. The combination of MXene’s unique physicochemical properties, including high electrical conductivity, large specific surface area, tunable RI, and abundant surface terminations, with the inherent advantages of optical fibers enables highly sensitive, label-free, and miniaturized sensing platforms. In general, MXene-based optical fiber sensors can be classified into two main categories according to their target analytes: chemical sensing, which focuses on detecting environmental or physicochemical parameters such as RI, salinity, ions, gases, and organic solvents, and biological sensing, which encompasses the detection of biomarkers, microorganisms, and physiological parameters (such as temperature and RH) that are closely associated with biological or medical processes. The following subsections summarize the representative demonstrations of these two application domains, highlighting the sensing mechanisms, performance metrics, and enhancement strategies enabled by MXene integration.

## Chemical Sensing Applications

### Refractive Index and Salinity Sensing

Although RI is a fundamental parameter in many biological detection schemes, in this review, it is categorized under chemical sensing because RI variation reflects the optical response of the surrounding medium rather than the direct recognition of biological species. RI sensors are often used as baseline platforms to characterize the intrinsic optical performance and sensitivity of MXene-enhanced devices before functionalization for specific biochemical detection. One of the earliest experimental demonstrations of MXene-enhanced optical fiber sensors involved the deposition of MXene nanosheets onto heterostructure fiber devices (MMF–SMF–MMF) for RI sensing in 2020,^72^ as illustrated in Figure 8a. Two sensing mechanisms were investigated in this work: direct evanescent-field-based transmission measurement and SPR-based spectral shift monitoring. Device samples with different MXene coating thicknesses were fabricated using optical deposition techniques with MXene dispersions of varying concentrations. Figure 8b shows SEM images of fiber surfaces coated with different MXene thicknesses; in one case, the coating was discontinuous, while in another, a uniform film approximately 5 μm thick was observed. Heterostructure devices with and without MXene coatings were tested under different ambient refractive indices, as shown in Figure 8c. Sensitivity enhancements of 6.1- and 8.9-fold were achieved for the two different coating thicknesses. For the SPR-based configuration, Figure 8d illustrates the spectral characteristics of devices with increasing MXene thicknesses, where a distinct red shift of the resonance wavelength was observed. The RI sensitivity increased correspondingly with MXene thickness, reaching a maximum enhancement of approximately 30%. However, it is worth noting that the fwhm of the resonance spectrum also broadened with increasing MXene thickness due to additional optical losses, which could potentially reduce the FOM, a trade-off not explicitly analyzed in the original study. Nevertheless, this work experimentally verified the potential of MXenes as a promising functional layer for sensitivity enhancement across different types of optical fiber sensors. Following this foundational work, the same group extended their research to salinity sensing. In 2022, a Ti_3_CNT_*x*_ MXene-coated interferometric sensor was developed, achieving an ultrahigh sensitivity of −5.34 nm/‰ over a 0–5‰ salinity range.^43^ A detailed study of the adsorption spectrum of Ti_3_CNT_*x*_, its effect on the interferogram, and the dependence of sensitivity on coating thickness confirmed that MXene integration significantly amplified light–matter interaction through enhanced evanescent-field coupling. Further improvements were demonstrated using an OMC operating near the dispersion turning point. Deposition of Ti_3_C_2_T_*x*_ MXene on the tapered region yielded an experimentally measured sensitivity of −1.7 × 10^5^ nm/RIU,^41^ positioning it among the most responsive fiber-based RI sensors reported to date and an excellent candidate for subsequent biosensing functionalization.

**Figure 8:**
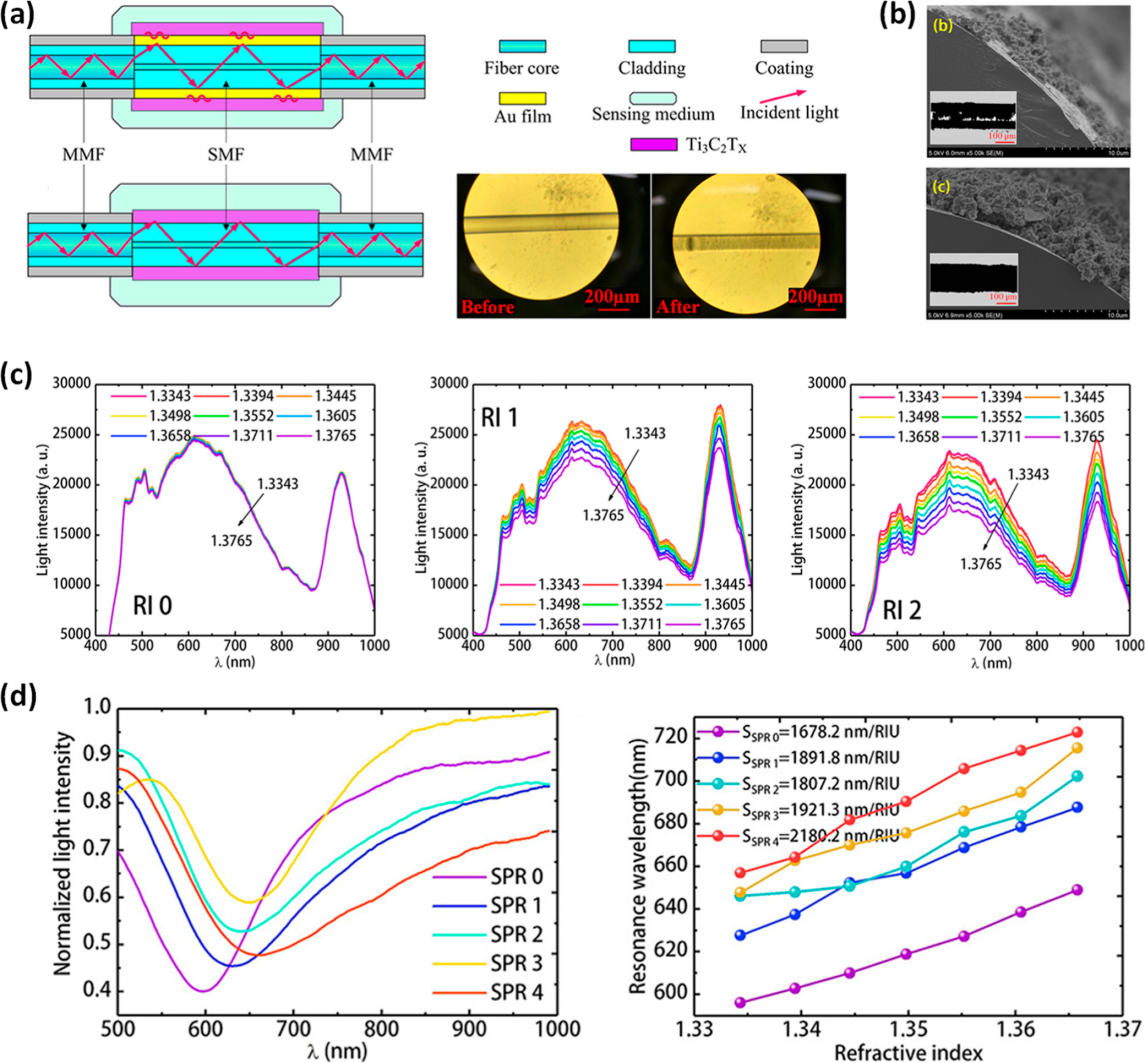
One of the first experimental demonstrations of MXene-based optical fiber sensors for RI sensing with enhanced sensitivity. (a) Structural diagram of the two proposed sensor configurations based on SPR and evanescent-field sensing; microscopy images of a representative device before and after MXene deposition are also shown. (b) SEM images of fiber surfaces coated with different MXene thicknesses using two dispersion concentrations. (c) Transmission spectra of the evanescent-field sensors in response to varying RIs. “RI 0” denotes the uncoated device, while “RI 1” and “RI 2” represent devices with increasing MXene thickness, where “RI 2” corresponds to a uniform film approximately 5 μm thick. (d) SPR spectra of devices with different MXene coating thicknesses and their responses to RI variation. “SPR 0” refers to the uncoated device, whereas “SPR 4” corresponds to the thickest MXene coating. Adapted with permission from ref 72. Copyright 2020 American Chemical Society.

Beyond interferometric platforms, several SPR-based configurations have been explored for RI sensing enhancement via MXene coatings. In 2022, a D-shaped fiber SPR sensor coated with Ti_3_C_2_T_*x*_ MXene via spin-coating exhibited a 53.6% improvement in RI sensitivity compared to the uncoated device.^88^ Similarly, an arc-shaped fiber SPR sensor utilizing Ti_2_CT_*x*_ MXene showed significant enhancement, with sensitivity increasing from 1601.9 nm/RIU for bare gold to 3652.5 nm/RIU at an optimal MXene concentration of 15 mg/mL.^91^ These improvements were attributed to MXene’s high surface area, hydrophilic terminations, and wide-band optical absorption, which collectively strengthened evanescent coupling at the metal–dielectric interface. In addition, an SPR sensor based on a silver-coated V-shaped photonic crystal fiber was reported in 2024.^87^ Deposition of a Ti_3_C_2_T_*x*_ MXene layer atop the silver film not only enhanced the RI sensitivity to 20,188 nm/RIU but also protected the silver layer from oxidation, thereby improving stability. By integrating a PDMS overlayer to exploit its large thermo-optic coefficient, the same platform achieved a high temperature sensitivity of 9.33 nm/°C, illustrating the versatility of MXene-modified plasmonic coatings. More recently, in 2025, a multilayer MXene/SnO_2_/Au structure was developed on an MMF–SMF–MMF fiber configuration for alcohol concentration measurement.^105^ The hybrid composite achieved a sensitivity of 3648.47 nm/RIU and exhibited excellent linearity (*R* ^2^ = 0.9953) and repeatability (<1.83% deviation over five cycles), underscoring the synergistic benefits of MXene–oxide–metal heterostructures for RI and chemical sensing applications. Collectively, these studies demonstrate that MXene integration, either as a standalone coating or within hybrid plasmonic composites, substantially enhances RI sensitivity, improves field confinement, and provides protective functionality for metallic films. These results establish MXene as a versatile and high-performance material platform for developing next-generation optical fiber chemical sensors.

### Ion, Liquid-Phase, and Gas-Phase Chemical Sensing

MXene-functionalized optical fiber sensors have been extensively explored for detecting ions, liquid-phase analytes, and gaseous species. Owing to their large surface area, abundant surface terminations, and strong affinity toward metal cations and polar molecules, MXenes enable selective adsorption and charge transfer at the fiber–material interface, thereby amplifying RI-induced spectral variations in the presence of trace analytes.

Photothermal chemical detection represents one of the earliest MXene-assisted optical fiber sensing applications. In 2021, a Nb_2_CT_*x*_ MXene-coated TFBG was used for organophosphorus pesticide detection through the molecular photothermal effect. Upon excitation, localized heating by the pesticide molecules induced RI changes that were captured by the cladding-mode spectrum of the TFBG. The MXene coating enhanced analyte adsorption and thermal conductivity, enabling temperature-compensated, high-sensitivity detection with a limit of detection (LoD) of 0.35 ppm.^106^ Building on this platform, the same group developed several MXene-enhanced photothermal sensors. In 2022, a Nb_2_CT_*x*_-integrated OMC enabled low-concentration antibiotic detection (streptomycin sulfate), achieving superior sensitivity compared with UV–vis and Raman spectroscopy methods. In 2023, a PDMS-modified Ti_3_CNT_*x*_ MXene sensor was fabricated for oxacillin sodium detection, in which the MXene layer facilitated molecular absorption while the PDMS coating, with its high thermo-optic coefficient, amplified the photothermal response.^55^ Combining the photothermal effect, high thermo-optic coefficient of PDMS, and enhanced absorption of molecules due to the MXene layer, the composite film coated device demonstrated improved performance compared to the bare device. A similar strategy was applied to a balloon-shaped fiber probe for antibiotic monitoring, achieving improved selectivity and stability through PDMS/MXene composite coatings.^49^

Beyond liquid-phase antibiotics and pesticides, MXene coatings have enabled highly sensitive ion detection. In 2023, a Ti_3_C_2_T_*x*_ MXene-coated LPG was used for rapid Cu^2+^ detection.^63^ The device achieved a sensitivity of 1.8 × 10^–4^ nm/ppb and an LoD of 25 ppb. Subsequent modification of the MXene layer with mussel-inspired poly levodopa improved the surface adsorption sites and hydrophilicity, enhancing both sensitivity (3.48 × 10^–4^ nm/ppb) and LoD (10.34 ppb). Similarly, in 2024, a Ti_2_CT_*x*_-MXene-coated arc-shaped SPR fiber was developed for Pb^2+^ detection.^104^ The MXene layer enriched the surface with oxygen- and hydroxyl-terminated groups that preferentially interacted with Pb^2+^ ions, yielding improved selectivity against competing metal ions.

MXene-based hybrid composites have also been explored for volatile chemical and gas-phase sensing. In 2023, Ti_3_C_2_T_*x*_ MXene/TiO_2_ hybrids were synthesized via liquid-phase deposition of (NH_4_)_2_TiF_6_ and coated onto U-shaped MMFs. The resulting devices exhibited an extraordinary RI sensitivity enhancement of 1056%, along with robust ammonia detection in both air and water, achieving a detection limit of 1.02 ppm and excellent selectivity over acetone, hexane, and methanol.^69^ Subsequently, similar U-shaped configurations employing electrospun polyacrylonitrile (PAN)/Ti_3_C_2_T_*x*_ MXene/TiO_2_ nanofiber coatings were developed for detecting dipolar aprotic solvent vapors such as *N*,*N*-dimethylformamide (DMF) and dimethyl sulfoxide (DMSO).^70,71^ The use of PAN nanofibers with highly polar cyano groups to modify the MXene coating demonstrated an effective increase in both sensitivity and selectivity, providing a promising route toward further functional film optimization. These composite films combined the large surface area and polarity of PAN with MXene’s high conductivity and TiO_2_’s surface reactivity, achieving a DMSO LoD of 3.2 ppm with excellent selectivity over other solvents. The synergistic dipole–dipole interactions and increased evanescent-field penetration within the nanofiber coating accounted for the enhanced sensitivity and rapid recovery.

In addition to interferometric and U-shaped configurations, the TFBG has also been investigated for MXene-enhanced chemical sensing, particularly in SPR applications.^102,103^ Compared with other SPR platforms, TFBG-based sensors exhibit stronger surface energy density owing to the synergistic coupling between the periodic modulation of the fiber core and the excitation of surface plasmon waves. This coupling produces an array of narrow cladding-mode resonances, yielding dense comb-like spectra with a high spectral resolution (≈0.1 nm). In one representative example, a Ta_2_CT_*x*_ MXene/gold film-coated TFBG sensor achieved an exceptionally high FOM of 2586 RIU^–1^.^102^ As illustrated in Figure 9a, the interfacial schematic shows the multilayer structure comprising the fiber core, the gold plasmonic film, and the MXene overlayer. Figure 9b displays an SEM image of the gold-coated fiber surface, confirming a uniform 50 nm gold film suitable for SPR excitation. The growth process of the MXene layer at different optical deposition times (50, 100, 150, and 200 min) is depicted in Figure 9c–f, revealing a progressive transition from scattered flakes to continuous membrane-like films. However, aggregation was observed at prolonged deposition times (200 min), which corresponded to a reduction in RI sensitivity and FOM, as shown in Figure 9g. The spectral responses of the sensor toward two carcinogenic food additives with different concentrations are presented in Figure 9h,i, demonstrating highly sensitive detection performance. These results confirm that controlling the MXene film thickness plays a crucial role in optimizing plasmonic coupling and that TFBG-based MXene-SPR structures provide a powerful and tunable platform for chemical sensing applications.

**Figure 9:**
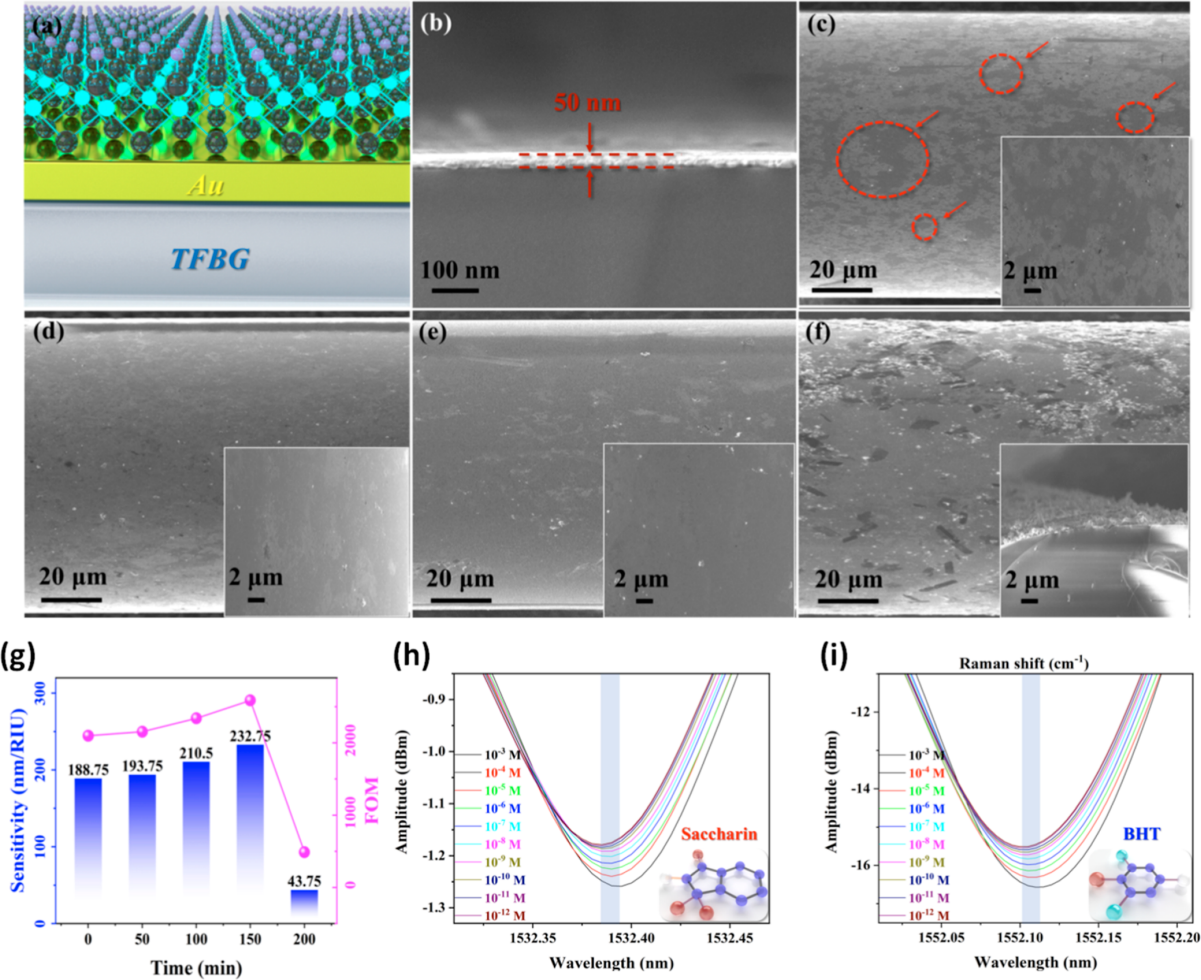
MXene-enhanced TFBG SPR sensor. Interfacial schematic of the Ta_2_CT_*x*_ MXene/Au/TFBG sensor structure. (b) Cross-sectional SEM image of the Au-coated TFBG. (cf) Surface morphologies of Ta_2_CT_*x*_ MXene/Au/TFBG sensors obtained at different deposition times (50–200 min; 50 min increments); the inset maps highlight the progressive growth state of the Ta_2_CT_*x*_ layer. (g) Sensitivity and FOM characteristics of sensors prepared with varying deposition durations. SPR spectra of Ta_2_CT_*x*_ MXene/Au/TFBG sensors in response to different concentrations (10^–12^–10^–3^ M) of (h) saccharin and (i) butylated hydroxytoluene (BHT). Adapted with permission from ref 102. Copyright 2022 Elsevier.

Table 4 summarizes representative MXene-functionalized optical fiber sensors and their reported performance in chemical sensing, including RI, salinity, ion, liquid-phase, and gas-phase detection. These studies reveal the versatility of MXene-based optical fiber sensors for detecting diverse chemical species. Through the integration of MXene with polymers or oxides, optical fibers gain enhanced adsorption capacity, thermal conductivity, and charge-transfer pathways, resulting in substantial improvements in sensitivity, selectivity, and response stability. The continuous advancement in MXene chemistry and coating engineering offers a robust foundation for developing next-generation environmental and industrial chemical sensors based on optical fibers.

**Table 4:** Representative MXene-Functionalized Optical Fiber Sensors and Their Performance in Chemical Sensing, Including RI, Salinity, Ion, Liquid-Phase, and Gas-Phase Detection

Sensing Type/Ref	Sensor Configuration + Demodulation	Sensitivity	Range	Limit of Detection
RI^72^	Heterostructure + Power	510.1%/RIU^a^	1.3343–1.3765	N.A
RI^72^	Heterostructure + SPR	2180.2 nm/RIU	1.3343–1.3658	N.A
RI^69^	U-shaped fiber + Power	1056%/RIU	1.33–1.36	N.A
RI^41^	OMC + Spectral shift	1.7 × 10^5^ nm/RIU	1.3330–1.3333	N.A
RI^88^	D-shaped fiber SPR + Spectral shift	3143 nm/RIU	1.3335–1.3541	N.A
RI^87^	V-shaped PCF^b^ SPR + Spectral shift	20188 nm/RIU	1.333–1.420	N.A
RI^91^	Arc-shaped fiber SPR + Spectral shift	3652.5 nm/RIU	1.330–1.343	N.A
RI^105^	Heterostructure SPR + Spectral shift	3648.47 nm/RIU	1.333–1.360	N.A
Salinity^43^	SMF + NCF + TNCF + NCF + SMF + Spectral shift	5.34 nm/‰	0–5‰	N.A
Ammonia^69^	U-shaped fiber + Power	N.A	0–25 ppm	1.02 ppm
*N*,*N*-Dimethylformamide^70^	U-shaped fiber + Power	2.3 mV/ppm	1–120 ppm	5.33 ppm
Dimethyl sulfoxide^71^	U-shaped fiber + Power	3.82 mW/ppm	0–698 ppm	3.2 ppm
Organophosphorus pesticides^106^	TFBG + Spectral shift	1.8 pm/ppm	10–50 ppm	0.38 ppm
Cu^2+^ ^63^	LPG + Spectral shift	3.48 × 10^–4^ nm/ppb	0–10^3^ ppb	10.34 ppb
Lead ions^104^	Arc-shaped fiber SPR + Spectral shift	14.81 nm/M	0–2.5 M	0.241 M
Streptomycin sulfate^40^	OMC + Spectral shift	210.66 nm/(mg/mL)	0.02–0.1 mg/mL	94.93 ng/mL
Oxacillin sodium^55^	Microfiber interferometer + Spectral shift	761.39 nm/(mg/mL)	0.02–0.1 mg/mL	26 ng/mL
Antibiotic^49^	Balloon-shaped fiber + Spectral shift	0.61 nm/(mg/L)	0–100 mg/L	0.0164 mg/L
Saccharin and BHT^102^	TFBG SPR + Spectral shift	N.A	10^–3^–10^–11^ M	10^–11^ M
Melamine^103^	TFBG SPR + Spectral shift	N.A	10^–2^–10^–11^ M	7.9 × 10^–9^ M

a Normalized transmission intensities.

b PCF: photonic crystal fiber.

## Biological Sensing Applications

While MXene-based optical fiber sensors have demonstrated outstanding performance in a variety of chemical sensing scenarios, including RI, salinity, and ion detection, the same material advantages that enable high chemical sensitivity also naturally translate to biological sensing. In biological environments, MXenes offer strong surface adsorption, excellent biocompatibility, and rich functional terminations for biomolecule immobilization, enabling highly sensitive interrogation of biological markers through RI modulation. Building upon the chemical sensing platforms discussed in Section 2, these MXene-functionalized fiber devices have been adapted for label-free biomarker detection, metabolic monitoring, and in situ biochemical assays, often through selective surface functionalization or nanocomposite engineering to achieve molecular specificity. The following subsections summarize representative advances in MXene-based optical-fiber biosensors for biomarker detection and physiological monitoring.

### Biomarker Detection and Biochemical Assays

In 2022, a Nb_2_CT_*x*_-coated tapered microfiber interferometer was reported for ultrasensitive hemoglobin detection using a light-controlled sensing scheme.^35^ However, the specificity of the device was not characterized against other relevant protein molecules. In 2024, a Ti_2_CT_*x*_-MXene-sensitized MZI was developed for in situ measurement of cytochrome *c* concentration, an important biomarker for apoptosis and mitochondrial dysfunction.^45^ The Ti_2_CT_*x*_ coating provided abundant surface functional groups that facilitated the immobilization of cytochrome *c* antibodies through electrostatic interactions, thereby amplifying the effective RI change in the sensing arm of the interferometer upon target binding. In addition, the layered structure and large specific surface area of the Ti_2_CT_*x*_ film enhanced evanescent-field penetration and ensured efficient light–analyte interaction at the fiber–MXene interface. These combined effects led to a pronounced improvement in sensing performance compared with the uncoated interferometer. In 2024, a Ti_3_C_2_T_*x*_ MXene@SGNps composite was integrated onto an optical fiber to monitor riboflavin secretion from Shewanella bacteria in situ.^44^ The gold nanoparticle array provided LSPR, while the MXene nanosheets acted as a conductive, high-surface-area scaffold facilitating charge transfer and strong molecular adsorption. This synergistic MXene–plasmonic hybrid significantly enhanced spectral modulation upon riboflavin binding. The device enabled real-time monitoring of riboflavin concentration during bacterial metabolic processes without the need for labeling or sample extraction, highlighting the potential of MXene–plasmonic composites for continuous biochemical assays in complex biological environments. Beyond metabolite detection, MXene-based fiber sensors have shown great potential for nucleic acid sensing. In 2025, an all-fiber device functionalized with Ti_3_C_2_T_*x*_ MXene and gold nanoparticles was demonstrated for detecting alkane degradation genes through a hybridization-induced RI change.^48^ The MXene film served as an efficient substrate for immobilizing single-stranded DNA probes due to its large surface area and abundant functional terminations, while gold nanoparticles enabled plasmonic enhancement and improved probe binding efficiency. Upon complementary DNA hybridization, the combined MXene–AuNPs (gold nanoparticle) coating produced strong optical modulation, enabling a low detection limit and high specificity against mismatch sequences. This platform exemplifies the adaptability of MXene-integrated fiber sensors for genotypic assays and environmental microbiological monitoring.

In addition to interferometric platforms, MXene-integrated SPR and LSPR fiber devices have been extensively explored for biosensing due to their ability to support strong field confinement and plasmonic coupling at the fiber–coating interface. Several representative MXene-assisted LSPR fiber sensors have been demonstrated In 2023, an Nb_2_CT_*x*_-modified double-S-tapered microfiber LSPR sensor was developed for tyramine detection, where the MXene coating served as a high-affinity adsorption layer and an efficient platform for immobilizing gold nanoparticles.^95^ The sensor exhibited improved LSPR peak shifts due to the stronger localized field and increased surface binding density. A related configuration based on a multicore-fiber (MCF) waist-expanded taper, enhanced by offset splicing and gold nanoparticle deposition, achieved ultrasensitive detection of Aflatoxin B1 across a broad dynamic range by leveraging efficient plasmon–waveguide coupling.^96^ WaveFlex-type biosensors, flexible and low-loss plasmonic fiber platforms, have also benefited from MXene integration. In 2023, Ti_3_C_2_T_*x*_-assisted WaveFlex fibers demonstrated highly selective histamine detection by exploiting the strong interaction between MXene nanosheets and the target analyte, along with enhanced LSPR coupling from immobilized gold nanostructures.^97^ In 2024, the concept was further expanded by incorporating Fe_3_O_4_–chitosan/MXene composites onto MMF–MCF–MMF WaveFlex structures for the detection of doxorubicin, where the magnetic–polymer–MXene hybrid improved molecular capture and provided efficient interfacial charge transport.^98^ Likewise, MXene quantum-dot-functionalized TIT4T WaveFlex fibers enabled sensitive xanthine detection.^99^ Another MXene-assisted W-shaped fiber LSPR device demonstrated highly selective tyramine detection through optimized surface functionalization of MXene and Au nanoparticles, offering strong analyte discrimination.^100^ Beyond detection of small biomolecules, MXene-assisted LSPR sensors have also been extended toward complex biological targets. In 2023, an optical microfiber decorated with Ti_3_C_2_T_*x*_-supported gold nanorods (GNRs) enabled combined ultrasensitive detection of renal-cancer-associated carbonic anhydrase IX (CAIX) protein and live renal cancer cells.^94^ The Ti_3_C_2_T_*x*_/GNR hybrid layers produced intense localized fields in the telecommunication band, significantly amplifying RI changes induced by aptamer–protein binding. The sensor achieved an extraordinary limit of detection of 13.8 zM for CAIX protein in buffer and 0.19 aM in serum and also detected 786-O renal cancer cells with a limit of 180 cells/mL. This work demonstrated the feasibility of MXene-supported plasmonic microfibers as powerful platforms for high-fidelity cancer biomarker and cell analysis.

In terms of SPR devices, early demonstrations include Ti_3_C_2_T_*x*_-based fiber SPR biosensors for growth differentiation factor 11 (GDF11) detection in 2021, where the MXene coating enhanced both sensitivity and spectral contrast relative to bare gold-film devices.^101^ Subsequent work expanded these designs using more sophisticated multilayer architectures and composite films and extended MXene SPR sensing to a variety of clinically relevant biomarkers. In 2024, a Ti_3_C_2_T_*x*_ MXene/AuNPs composite film was integrated onto a D-shaped fiber to realize label-free detection of human IgG.^89^ In 2025, Ti_3_C_2_T_*x*_ MXene was further applied to develop an SPR fiber biosensor for detecting *Staphylococcus aureus*, wherein MXene’s abundant surface groups facilitated stable immobilization of specific antibodies.^90^ The device exhibited a clear and monotonic spectral response to bacterial concentration with high specificity against interfering species. The MXene-enhanced SPR framework has also proven effective for detecting growth factors. An SPR biosensor based on a heterostructure architecture, functionalized with Ti_3_C_2_T_*x*_–polydopamine (PDA) composites, enabled ultrasensitive detection of placental growth factor in 2025.^93^ The MXene–PDA hybrid improved biomolecule capture efficiency while preserving excellent optical coupling, yielding a low detection limit suitable for early-stage diagnostics of pregnancy-related disorders. Particularly noteworthy is the 2025 Ti_3_C_2_T_*x*_ MXene/GNRs synergistically enhanced acetylcholine biosensor.^92^ Using an electrostatic layer-by-layer assembly method, Ti_3_C_2_T_*x*_ MXene nanosheets were first deposited onto a gold film, followed by GNRs with controlled aspect ratios. This hybrid configuration exploits the strong evanescent-field extension provided by MXene together with intense localized field “hot spots’’ formed at GNR tips, producing a synergistic plasmonic enhancement rather than a simple additive effect. As a result, the RI sensitivity improved by 217.7%, and the device achieved a detection sensitivity of 0.04521 nm/μM with an LoD of 4.42 μM for acetylcholine. This work highlights the powerful combination of 2D MXene layers and anisotropic plasmonic nanoparticles for next-generation biosensing.

Table 5 provides a summary of representative MXene-enhanced optical fiber interferometer, SPR, and LSPR biosensors, covering analytes ranging from small biomolecules to proteins, nucleic acids, and pathogenic bacteria. These studies reveal the significant potential of MXene-functionalized sensors for high-performance biosensing. By leveraging MXene’s tunable surface chemistry, broadband optical absorption, and strong plasmonic coupling with noble-metal nanostructures, these platforms achieve enhanced field confinement, improved biorecognition efficiency, and ultralow detection limits.

**Table 5:** Representative MXene-Functionalized Optical Fiber Sensors and Their Performance in Biomarker Detection and Biochemical Assays

Sensing Type/Ref	Sensor Configuration	Sensitivity	Range	Limit of Detection
Hemoglobin^35^	Microfiber	7.581 nm/(g/dL)	0–13 g/dL	0.0026 g/dL
Cytochrome *c* ^45^	MZI	1.428 nm/μM	0–7.04 μM	0.392 μM
Riboflavin^44^	MZI	5.61 nm/μM	0–2.656 μM	115 nM
Alkane degradation genes^48^	MZI	0.9 nm/nM	1 fM–1 μM	1 fM
Tyramine^95^	Double S-tapered fiber + LSPR	34 pm/μM	0–300 μM	0.35 μM
Aflatoxin B1 ^96^	MMF–MCF–MCF–MMF + LSPR	11.7 nm/μM	0–1000 nM	26.41 nM
Histamine^97^	WaveFlex + LSPR	4.4 pm/μM	0–1000 μM	52.5 μM
Doxorubicin^98^	WaveFlex + LSPR	0.885 nm/μM	0–10 μM	0.37 μM
Xanthine^99^	WaveFlex + LSPR	1.93 nm/μM	0–800 μM	146.11 μM
Tyramine^100^	WaveFlex + LSPR	0.0385 nm/μM	0–100 μM	6.96 μM
Renal cancer proteins and cells^94^	Microfiber + LSPR	1.46 nm/lg(M)	10^–20^–10^–10^ M	13.8 zM
GDF11 ^101^	Plastic clad fiber + SPR	8.49 nm/lg(g/L)	10^–11^–10^–6^ g/L	0.577 pg/L
IgG^89^	Heterostructure + SPR	1.7046 nm/(μg/mL)	0–30 μg/mL	0.17 μg/mL
Staphylococcus aureus^90^	Heterostructure + SPR	3.063 nm/log(CFU/mL)	10^2^–10^8^ CFU/mL	1.14 CFU/mL
Placental growth factor^93^	Heterostructure + SPR	12.313 nm/log(pg/mL)	1 pg/mL^–1^ ng/mL	1.354 pg/mL
Acetylcholine^92^	Y-type bifurcated optical fiber + SPR	0.04521 nm/μM	0–700 μM	4.42 μM

### Physiological and Respiratory Monitoring

Breath is one of the most important physiological indicators of human life, providing real-time information about the respiratory rhythm, metabolic activity, and overall health status.^107^ MXene-based optical fiber humidity sensors have shown great promise for respiratory monitoring, where RH variations correspond to the moisture content of exhaled air. The intrinsic hydrophilicity and layered structure of MXenes provide abundant active sites for water-molecule adsorption through hydrogen bonding and interlayer diffusion. These interactions modulate the effective RI and optical absorption of the MXene coating, which can be detected through spectral shifts or transmission variations in interferometric or evanescent-field fiber sensors. In 2022, based on a microfiber interferometer device with a waist diameter of 4.7 μm, a high-sensitivity RH sensor was developed by depositing Nb_2_CT_*x*_ nanosheets onto the sensing region of the interferometer.^52^ Nonmonotonic spectral responses were observed due to the different adsorption behaviors of the MXene coating under various RH levels. The response time, reported as 1.72 s, although faster than other optical fiber RH sensors based on different functional materials, was still not fast enough for real-time breath sensing. In 2024, a microfiber fabricated by chemical etching was coated with Ti_3_C_2_T_*x*_ MXene via optical deposition for RH sensing.^67^ The device exhibited a rapid response of 0.176 s and a recovery time of 0.521 s. Real-time breath monitoring was successfully demonstrated based on transmission-power measurements, showcasing the potential of this configuration for precise tracking of breath patterns. However, the effect of coating thickness on the device’s performance was not comprehensively investigated, leaving room for further optimization. In 2025, a similar microfiber coated with V_4_C_3_T_*x*_ MXene was reported for RH sensing.^68^ Microfibers with different diameters (15–18 μm) were investigated, revealing that the 17 μm diameter device provided the most repeatable performance and lowest hysteresis during both RH rising and falling processes within the 30–80% range. The response and recovery times were 0.125 and 0.386 s, respectively, showing clear potential for real-time breath monitoring. Interestingly, the observed trend of optical transmission differed from that in ref^67^, primarily due to the use of a different MXene composition, though the underlying mechanism remains to be clarified. A similar investigation was conducted on Ti_3_C_2_T_*x*_-coated microfibers with waist diameters ranging from 2 to 10 μm.^53^ The 4 μm device delivered the highest sensitivity (0.1482 dB/%RH) with good linearity, although the response time was not reported.

In addition to simple microfiber geometries, tapered NCFs have been widely employed to further enhance the evanescent-field coupling. In 2024, a tapered NCF sandwiched between two SMFs and coated with Ti_3_C_2_T_*x*_ MXene was demonstrated for RH sensing.^36^ A nonmonotonic spectral response was again observed, attributed to distinct dielectric behaviors of the MXene film under different humidity levels. A U-shaped tapered NCF configuration was subsequently proposed,^37^ providing a stronger evanescent field and markedly higher sensitivity. When packaged inside a face mask, the sensor enabled the accurate and real-time tracking of human breathing, confirming its practical capability for wearable respiratory monitoring. In parallel, a TFBG device functionalized with a nanoporous composite film comprising Ti_2_CT_*x*_ MXene and fullerene was developed for respiratory RH sensing.^64^ Although exhibiting longer response and recovery times, the TFBG device offered high mechanical robustness and inherent temperature self-compensation by utilizing the core-mode resonance, making it suitable for quasi-dynamic RH monitoring in variable environments.

Temperature is another vital physiological parameter closely associated with human health. Although MXene-based optical fiber temperature sensors were originally designed for industrial or environmental use, their fast response and compact configurations make them promising for real-time body temperature or breath temperature monitoring. In 2023, a Ti_3_C_2_T_*x*_-coated microstructured fiber MZI sensor was reported.^46^ The integration of the MXene coating improved the temperature sensitivity by 94.7%, from 61.99 pm/°C to 120.71 pm/°C. Compact resonant configurations, such as MKRs coated with V_2_CT_*x*_ MXene, were also investigated.^38,39^ MKRs with varying diameters and coating thicknesses were systematically studied, achieving a highest sensitivity of 0.32 dB/°C with excellent repeatability and stability. Furthermore, a fiber-tip FPI cavity filled with the Ti_3_C_2_T_*x*_/PDMS composite was developed for fast-response, high-sensitivity temperature sensing.^51^ By combining the small footprint of the FPI, the high thermal conductivity of MXene, and the large thermo-optic coefficient of PDMS, the hybrid system exhibited a temperature sensitivity of 31.62 nm/°C with a response time of 0.721 s.

Table 6 summarizes the performance of the MXene-enhanced optical fiber RH and temperature sensors. These studies demonstrate the great potential of MXene-based optical fiber sensors for physiological monitoring. Their high humidity and temperature sensitivities, rapid response times, and compatibility with compact or flexible fiber configurations offer an attractive route toward real-time, noninvasive monitoring of breath and thermal activity, paving the way for future integration into wearable and clinical diagnostic systems.

**Table 6:** Representative MXene-Functionalized Optical Fiber Sensors and Their Performance in RH and Temperature Measurements

Sensing Type/Ref	Sensor Configuration + Demodulation	Response/Recovery Time (s)	Sensitivity	Range	Sensor Package
RH^52^	Microfiber + Spectral shift	1.72	–86 pm/%RH585 pm/%RH	18.5–72.4%72.4–95.4%	No
RH^67^	Microfiber + Power	0.176/0.521	N.A	30–80%	No
RH^68^	Microfiber + Power	0.125/0.386	3.38 μW/%RH	30–80%	No
RH^64^	TFBG + Power	0.59/0.91	0.055 dB/%RH	10–90%	No
RH^53^	Microfiber + Power		0.1482 dB/%RH	25–60%	No
RH^36^	Tapered NCF + Spectral shift	3.5/12.7	–77 pm/%RH685 pm/%RH	33–75%75–91%	No
RH^37^	U-shaped tapered NCF + Spectral shift	0.025/0.12	0.44 nm/%RH1.11 nm/%RH	30–45%45–80%	Yes
Temperature^46^	SMF–NCF–MMF–NCF–SMF + Spectral shift	N.A	120.71 pm/°C	30–80 °C	No
Temperature^47^	SMF–NCF–MMF–NCF–SMF + Spectral shift	N.A	64 pm/°C	30–80 °C	No
Temperature^39^	MKR + Power	N.A	0.32 dB/°C	25–70 °C	No
Temperature^51^	FPI + Spectral shift	0.721	31.62 nm/°C	36.5–37.2 °C	No

## Conclusion and Perspectives

MXenes have rapidly emerged as one of the most powerful classes of functional materials for advancing optical fiber chemical and biosensing. Their unique combination of large specific surface area, abundant and tunable surface terminations, hydrophilicity, high electrical conductivity, and broad optical absorption window creates a highly responsive interface for light–matter interaction. When integrated with optical fiber platforms, whether interferometric, grating-based, SPR/LSPR-enhanced, or evanescent-field devices, MXenes consistently amplify optical responses. As demonstrated across a wide range of applications reviewed in this work, MXene coatings and MXene-based nanocomposites enable substantial improvements in sensitivity, dynamic range, detection limits, and selectivity for diverse chemical and biological analytes. These advances firmly establish MXene-functionalized optical fibers as a promising material–device pairing for next-generation, miniaturized, and highly responsive sensing systems.

The sensing functionality of MXenes can be viewed within a broader historical context that includes both metal-oxide (MOX) sensors and metal–organic frameworks (MOFs).^3,108,109^ Traditional MOX sensors rely on the modulation of electronic conductivity arising from adsorption-induced charge transfer and surface redox processes, enabling strong and direct transduction of chemical interactions into measurable electrical signals. MOFs, in contrast, were initially developed as high-surface-area, porous materials optimized for selective adsorption and molecular concentration, with sensing responses often dominated by dielectric or mass-loading effects. More recently, considerable effort has been directed toward the development of conductive MOFs, where guest adsorption can influence charge transport pathways and thereby enhance signal transduction.^110,111^ MXenes naturally combine features of both classes: like MOFs, they provide strong adsorption and a local concentration of analytes at the sensing interface, while like MOX materials, they possess delocalized charge carriers whose density and mobility can be reversibly modified by molecular adsorption. This intrinsic coupling between surface chemistry and electronic conductivity allows MXenes to strongly perturb electromagnetic fields, enabling efficient transduction across a wide frequency range, from microwave and radio frequency resonators to optical and plasmonic platforms. As such, MXenes occupy a distinct and complementary position in the landscape of sensing materials, bridging adsorption-driven selectivity with conductivity-driven signal amplification.

Despite these advances, several scientific and technological challenges remain before MXene-enhanced optical fiber sensors can reach their full maturity. One fundamental issue is oxidation stability, particularly for Ti_3_C_2_T_*x*_, which undergoes structural degradation under humid or oxygen-rich conditions.^112^ Moisture-induced oxidation alters electronic and optical properties, disrupts interlayer spacing, and compromises long-term sensing repeatability. Although recent studies, including surface passivation strategies, antioxidant additives, low-temperature curing, and encapsulation, have shown promise, achieving oxidation-resistant MXene films that retain strong optical activity remains a central objective.^113,114^ In addition, storage conditions, such as temperature, humidity, and exposure to air, must be carefully controlled to minimize degradation and ensure a stable device performance over extended periods. Another key challenge lies in the controlled fabrication of MXene coatings on the cylindrical fiber surfaces. Uniformity, thickness precision, and adhesion are critical for ensuring predictable changes in the effective RI, low optical scattering loss, mechanical stability, and reproducibility across devices. Nonuniform coatings or poor adhesion can introduce variations in optical coupling conditions, increase insertion loss, and ultimately lead to device-to-device performance inconsistency. While optical deposition, surface functionalization, and drop-casting techniques offer complementary advantages, each presents limitations in terms of film uniformity, process repeatability, or long-term adhesion. Furthermore, although MXene integration often improves sensitivity, higher sensitivity does not always mean better overall sensor performance. Excessive MXene thickness could introduce larger insertion loss, spectral broadening, hysteresis, and reduced stability. Therefore, MXene-based optical fiber sensors should be optimized by considering multiple performance metrics together, including sensitivity, FOM, resolution, stability, response speed, and reproducibility. Future work must explore more deterministic coating strategies, such as atomic-layer deposition-inspired approaches,^115^ controlled interlayer assembly,^116^ or ink-engineered MXene formulations,^117^ to reliably produce compact, homogeneous, and application-specific MXene films with improved reproducibility and long-term sensing stability.

From the materials perspective, beyond-Ti_3_C_2_T_*x*_ MXene chemistries represent an important frontier. Recent progress in synthesizing V_2_CT_*x*_, Nb_2_CT_*x*_, Ti_2_CT_*x*_, Mo_2_CT_*x*_, and ordered double-MXenes expands the available optical and electronic design space.^118^ As highlighted in recent MXene reviews,^119^ modifying surface terminations, engineering Janus MXenes, and constructing MXene–polymer, MXene–MOF, and MXene–semiconductor composites provide pathways to enhance selectivity, suppress oxidation, and tailor optical constants for targeted photonic responses. Such tunability will be central to building optical fiber biosensors with molecular-level specificity and robust performance under complex environmental conditions. On the device side, there is significant potential for multimodal and multiplexed sensing architectures. Hybrid platforms that combine interferometric transduction with plasmonic enhancement, MOF- or polymer-mediated selectivity, or machine-learning-based spectral decoding can push detection limits far below those achievable using any single mechanism. The possibility of integrating MXenes with distributed fiber sensing, e.g., leveraging Rayleigh, Brillouin, and Raman scattering or multiplexed FBGs, also opens the door to MXene-assisted distributed chemical mapping over multimeter scales, a capability largely unexplored to date.

Looking forward, MXene-enhanced optical fiber sensors are poised to play an important role in precision medicine, environmental surveillance, industrial-process monitoring, and intelligent sensing networks. Achieving this vision will require coordinated advances in MXene synthesis, surface engineering, composite design, thin-film processing, and fiber–material integration. Nevertheless, the progress reviewed in this work demonstrates that MXenes offer a uniquely powerful combination of tunable chemistry and strong optical responsiveness, positioning them as a cornerstone material for the next generation of high-performance, scalable, and intelligent optical fiber chemical and biosensing technologies.

## References

[1] Liu X., Huang D., Lai C., Zeng G., Qin L., Wang H., Yi H., Li B., Liu S., Zhang M. (2019). Recent advances in covalent organic frameworks (COFs)
as a smart sensing material. Chem. Soc. Rev..

[2] Wang H., Ma J., Zhang J., Feng Y., Vijjapu M. T., Yuvaraja S., Surya S. G., Salama K. N., Dong C., Wang Y. (2021). Gas sensing
materials roadmap. J. Phys.: Condens. Matter.

[3] Kreno L. E., Leong K., Farha O. K., Allendorf M., Van Duyne R. P., Hupp J. T. (2012). Metal-organic framework materials
as chemical sensors. Chem. Rev..

[4] Zhang H., Chhowalla M., Liu Z. (2018). 2D nanomaterials: graphene and transition metal dichalcogenides. Chem. Soc. Rev..

[5] Murali A., Lokhande G., Deo K. A., Brokesh A., Gaharwar A. K. (2021). Emerging
2D nanomaterials for biomedical applications. Mater. Today.

[6] Bhati V. S., Kumar M., Banerjee R. (2021). Gas sensing
performance of 2D nanomaterials/metal oxide nanocomposites: A review. J. Mater. Chem. C.

[7] Wang L., Xiong Q., Xiao F., Duan H. (2017). 2D nanomaterials based
electrochemical biosensors for cancer diagnosis. Biosens. Bioelectron..

[8] Liu B., Zhou K. (2019). Recent progress on
graphene-analogous 2D nanomaterials: Properties, modeling and applications. Prog. Mater. Sci..

[9] Gogotsi, Y. ; Anasori, B. The rise of MXenes. In MXenes; Jenny Stanford Publishing, 2023; pp 3–11.

[10] Naguib, M. ; Kurtoglu, M. ; Presser, V. ; Lu, J. ; Niu, J. ; Heon, M. ; Hultman, L. ; Gogotsi, Y. ; Barsoum, M. W. Two-dimensional nanocrystals produced by exfoliation of Ti3AlC2. In MXenes; Jenny Stanford Publishing, 2023; pp 15–29. 10.1002/adma.201102306 21861270

[11] Lim K. R. G., Shekhirev M., Wyatt B. C., Anasori B., Gogotsi Y., Seh Z. W. (2022). Fundamentals
of MXene synthesis. Nat. Synth..

[12] Pei Y., Zhang X., Hui Z., Zhou J., Huang X., Sun G., Huang W. (2021). Ti3C2TX MXene
for sensing applications: recent progress, design principles, and
future perspectives. ACS Nano.

[13] Sinha A., Dhanjai, Zhao H., Huang Y., Lu X., Chen J., Jain R. (2018). MXene: An emerging material for sensing
and biosensing. TrAC, Trends Anal. Chem..

[14] Ho D. H., Choi Y. Y., Jo S. B., Myoung J. M., Cho J. H. (2021). Sensing with MXenes: progress and
prospects. Adv. Mater..

[15] Li Y., Huang S., Peng S., Jia H., Pang J., Ibarlucea B., Hou C., Cao Y., Zhou W., Liu H. (2023). Toward smart sensing by MXene. Small.

[16] Wu L., Yuan X., Tang Y., Wageh S., Al-Hartomy O. A., Al-Sehemi A. G., Yang J., Xiang Y., Zhang H., Qin Y. (2023). MXene sensors
based on optical and electrical sensing signals: from biological,
chemical, and physical sensing to emerging intelligent and bionic
devices. PhotoniX.

[17] Wang Y., Wang Y., Jian M., Jiang Q., Li X. (2024). MXene key composites: a new arena for gas sensors. Nano-Micro Lett..

[18] Lu P., Lalam N., Badar M., Liu B., Chorpening B. T., Buric M. P., Ohodnicki P. R. (2019). Distributed
optical fiber sensing: Review and perspective. Appl. Phys. Rev..

[19] Zhao Y., Tong R.-j., Xia F., Peng Y. (2019). Current status of optical fiber biosensor based on surface plasmon
resonance. Biosens. Bioelectron..

[20] Xue X., Han X., Li W., Li K., Liu F., Guo T. (2024). Operando Battery Monitoring: Lab-on-Fiber Electrochemical Sensing
Technologies. Laser Photonics Rev..

[21] Polynkin P., Polynkin A., Peyghambarian N., Mansuripur M. (2005). Evanescent field-based optical fiber sensing device
for measuring the refractive index of liquids in microfluidic channels. Opt. Lett..

[22] Zhang W., Lang X., Liu X., Li G., Singh R., Zhang B., Kumar S. (2023). Advances in tapered optical fiber
sensor structures: from conventional to novel and emerging. Biosensors.

[23] Ying Y., Si G.-y., Luan F.-j., Xu K., Qi Y.-w., Li H.-n. (2017). Recent research progress of optical
fiber sensors based on D-shaped structure. Opt.
Laser Technol..

[24] Tan A. J. Y., Ng S. M., Stoddart P. R., Chua H. S. (2021). Trends and applications of U-shaped fiber optic sensors:
a review. IEEE Sens. J..

[25] Li X., Chen N., Zhou X., Gong P., Wang S., Zhang Y., Zhao Y. (2021). A review of
specialty fiber biosensors based on interferometer configuration. J. Biophotonics.

[26] Wu Q., Qu Y., Liu J., Yuan J., Wan S.-P., Wu T., He X.-D., Liu B., Liu D., Ma Y. (2021). Singlemode-Multimode-Singlemode Fiber Structures for Sensing Applications—A
Review. IEEE Sens. J..

[27] Pacheco-Peña V., Hallam T., Healy N. (2022). MXene supported
surface plasmons on telecommunications optical fibers. Light:Sci. Appl..

[28] Shahzad F., Zaidi S. A., Naqvi R. A. (2022). 2D transition
metal carbides (MXene) for electrochemical sensing: A review. Crit. Rev. Anal. Chem..

[29] Rhouati A., Berkani M., Vasseghian Y., Golzadeh N. (2022). MXene-based electrochemical sensors for detection of
environmental pollutants: A comprehensive review. Chemosphere.

[30] Mathew M., Rout C. S. (2021). Electrochemical
biosensors based on Ti3C2Tx MXene: future perspectives for on-site
analysis. Curr. Opin. Electrochem..

[31] Zhu C., Gerald R. E., Huang J. (2020). Progress toward sapphire optical
fiber sensors for high-temperature applications. IEEE Trans. Instrum. Meas..

[32] Elsherif M., Salih A. E., Muñoz M. G., Alam F., AlQattan B., Antonysamy D. S., Zaki M. F., Yetisen A. K., Park S., Wilkinson T. D. (2022). Optical fiber sensors: Working principle, applications, and limitations. Adv. Photonics Res..

[33] Lee B. H., Kim Y. H., Park K. S., Eom J. B., Kim M. J., Rho B. S., Choi H. Y. (2012). Interferometric fiber optic sensors. Sensors.

[34] Korposh S., James S. W., Lee S.-W., Tatam R. P. (2019). Tapered optical
fibre sensors: Current trends and future perspectives. Sensors.

[35] Li W., Miao Y., Zheng Y., Zhang K., Yao J. (2022). Nb 2 CT x MXene integrated tapered
microfiber based on light-controlled light for ultra-sensitive and
wide-range hemoglobin detection. IEEE Sens.
J..

[36] Liu P., Feng L., Chen H., Li P., Ma X., Lv M. (2024). Humidity sensor based on tapered
no-core fiber coated with Ti3C2Tx MXene. Ceram.
Int..

[37] Cong J., Yang M., Zhou D., Meng L., Feng S., Lv M. (2024). High sensitivity humidity sensor based on the U-shaped tapered no-core
fiber coated with MXene. Opt. Lett..

[38] Chen S., Ran J., Zheng T., Wu Q. (2023). Ultracompact MXene V2C-improved temperature sensor by a runway-type
microfiber knot resonator. Nanomaterials.

[39] Wu Q., Ran J., Zheng T., Wu H., Liao Y., Wang F., Chen S. (2023). MXene V 2 C-coated runway-type microfiber
knot resonator for an all-optical temperature sensor. RSC Adv..

[40] Zhang W., Miao Y., Zhang H., Yao J. (2022). Low-concentration antibiotic
detection in water based on enhanced photothermal effect. Appl. Phys. Lett..

[41] Peng R., Zhou W., Wu Y., Song Z., Yu H. (2024). Ultrasensitive refractive index sensing
of optical microfiber couplers coated with Ti3C2MXene. Opt. Mater. Express.

[42] Li K., Zhang T., Liu G., Zhang N., Zhang M., Wei L. (2016). Ultrasensitive optical microfiber coupler based sensors operating
near the turning point of effective group index difference. Appl. Phys. Lett..

[43] Yi D., Wang C., Gao L., Chen Y., Liu F., Geng Y., Zhang H., Li X. (2022). Ti3CN MXene-based ultra-sensitive optical fiber salinity sensor. Opt. Lett..

[44] Jin P., Zhou E., Cheng L., Zheng W., Zhao Y., Zhai S., Zhang Y. n. (2024). In Situ Label-Free Monitoring of
Riboflavin Concentration in Shewanella via a Fiber-Optic Biosensor
Modified by Ti3C2-MXene@ SGNps Composites. Adv.
Opt. Mater..

[45] Jin P., Zhang Y.-n., Li Z., Zheng W., Cheng L., Li L., Li X., Zhao Y. (2024). In-situ and label-free measurement of cytochrome C concentration
with a Ti2C-MXene sensitized fiber-optic MZI sensor. Anal. Chim. Acta.

[46] Wu Y., Feng Y., Liu X., Shen T., Zhang H. (2023). Intrinsic MXene-Ti3C2Tx enhanced
high sensitivity Mach-Zehnder interferometric microstructured optic
fiber temperature sensor. Opt. Fiber Technol..

[47] Zhou C., Liu X., Feng Y., Shen T. (2025). High sensitivity
Mach-Zehnder optic fiber temperature sensor based on V2C-MXene sensitization. Opt. Commun..

[48] Song Q., Huang Z., Xiao Y., Zhang Y., Li L., Yu Y., Chen S., Wu Q. (2025). MXene Ti_3_C_2_-AuNPs based all-fiber device for
alkane degradation genes detection. Microchem.
J..

[49] Dai L., Huang W., Su C., Tuz V. R., Geng T. (2025). Probe-based fiber sensor with PDMS/Mxene
coating technology for antibiotic detection. Opt. Laser Technol..

[50] Zhu C., Zheng H., Ma L., Yao Z., Liu B., Huang J., Rao Y. (2023). Advances in fiber-optic
extrinsic Fabry-Perot interferometric physical and mechanical sensors:
A review. IEEE Sens. J..

[51] Li P., Wang Z., Li H., Feng S., Meng L., Lv M. (2024). Development of a high-sensitivity
and fast-response fiber temperature sensor utilizing Ti3C2TX MXene/PDMS
composites and Vernier effect. Opt. Express.

[52] Bi M., Miao Y., Li W., Yao J. (2022). Niobium carbide MXene-optics fiber-sensor for high sensitivity humidity
detection. Appl. Phys. Lett..

[53] Mustaffa S. N., Menon P. S., Ab Razak M. Z., Izani M. H., Aminuddin
Jafry A. A., Harun S. W., Muhammad A. R. (2025). Tailoring Waist
Diameter of Tapered Optical Fiber Coated with MXene Ti3C2Tx For Enhanced
Relative Humidity Sensing. Fiber Integr. Opt..

[54] Li H., Yang K., Hu H., Qin C., Yu B., Zhou S., Jiang T., Ho D. (2024). MXene supported
surface plasmon polaritons for optical microfiber ammonia sensing. Anal. Chem..

[55] Zhang W., Zheng Y., Miao Y., Yao J. (2023). High-sensitivity
optical fiber photothermal sensor for antibiotic detection with PDMS/Ti
3 CN MXene composite coating. IEEE Sens. J..

[56] Broadway C., Min R., Leal-Junior A. G., Marques C., Caucheteur C. (2019). Toward commercial polymer fiber Bragg
grating sensors: Review and applications. J.
Lightwave Technol..

[57] Mihailov S. J. (2012). Fiber Bragg grating sensors for harsh environments. Sensors.

[58] Sahota J. K., Gupta N., Dhawan D. (2020). Fiber Bragg grating sensors for monitoring
of physical parameters: A comprehensive review. Opt. Eng..

[59] Esposito F., Srivastava A., Sansone L., Giordano M., Campopiano S., Iadicicco A. (2021). Label-free biosensors based on long period fiber gratings:
a review. IEEE Sens. J..

[60] Albert J., Shao L. Y., Caucheteur C. (2013). Tilted fiber
Bragg grating sensors. Laser Photonics Rev..

[61] Del Villar I., Fuentes O., Chiavaioli F., Corres J. M., Matias I. R. (2018). Optimized strain long-period fiber
grating (LPFG) sensors operating at the dispersion turning point. J. Lightwave Technol..

[62] Del Villar I. (2015). Ultrahigh-sensitivity sensors based on thin-film coated
long period gratings with reduced diameter, in transition mode and
near the dispersion turning point. Opt. Express.

[63] Yan M., Li Y., Li Y., Liu B., Wang R., Jiang M. (2023). Long period fiber grating sensor
coated with MXene (Ti3C2TX) functionalized by poly-levodopa for rapid
detection of Cu2+. Optik.

[64] Wu J., Shi Y., Fen X., Zhou J., Dong J., Zhou W. J., Shen C. (2024). Respiratory
monitoring via a nanoporous film-coated tilted fiber Bragg grating
humidity sensor. Opt. Lett..

[65] Alberto N. J., Marques C. A., Pinto J. L., Nogueira R. N. (2010). Three-parameter
optical fiber sensor based on a tilted fiber Bragg grating. Appl. Opt..

[66] Zhao P., Ho H. L., Fan S., Jin W. (2023). Evanescent Wave Lab-on-Fiber
for High Sensitivity Gas Spectroscopy with Wide Dynamic Range and
Long-Term Stability. Laser Photonics Rev..

[67] Li X., Sun B., Xue T., Pan K., Su Y., Jiang Y., Du B., Yang D. (2024). MXene-based fiber-optic
Humidity Sensor for Fast Human Breath Monitoring. Photonics.

[68] Ryu J., Woo T., Kim J., Jo J., Joe N., Kwon S.-y., Lee C.-K., Lee J. H. (2025). A relative
humidity sensor based on V4C3MXene-coated etched optical fiber. Opt. Quantum Electron..

[69] Wang T., Zhu L., Kanda H. (2023). Ti3C2MXene-TiO2
hybrid-modified U-bend fiberoptic sensor for improved refractive index
sensitivity and ammonia detection. Sens. Actuators,
B.

[70] Wang T., Zhu L., Yue Y., Asghari M. R., Samani B. H., Yamamoto T., Mukai Y., Kanda H. (2024). N, N-dimethylformamide detection and refractive index sensing using
an electrospun polymer/Ti3C2MXene-TiO2 modified optical fiber sensor. Sens. Actuators, B.

[71] Wang T., Zhu L., Yue Y., Mukai Y., Kanda H., Yamamoto T. (2025). Fiber-optic sensor
modified by electrospun Polymer/Ti3C2MXene-TiO2 for dimethyl sulfoxide
sensing. Talanta.

[72] Chen Y., Ge Y., Huang W., Li Z., Wu L., Zhang H., Li X. (2020). Refractive index sensors
based on Ti3C2Tx MXene fibers. ACS Appl. Nano
Mater..

[73] Liu Y., Li K., Wang R., Wang Y., Wang G., Meng X. (2025). A Highly Sensitive
D-Shaped Microstructured Fiber SPR Biosensor Based on MXene-Au-TiO2
Composite Film Coating. Plasmonics.

[74] Mao Y., Ren F., Zhou D., Li Y. (2025). Highly sensitive PCF-SPR RI sensor for cancer detection using gold/graphene/Ti3C2Tx-MXene
hybrid layer. Plasmonics.

[75] Uniyal A., Pal A., Ansari G., Chauhan B. (2025). Numerical simulation of InP and MXene-based SPR sensor
for different cancerous cells detection. Cell
Biochem. Biophys..

[76] Basha A. J., Maheshwari R. U., Pandey B. K., Pandey D. (2025). Ultra-sensitive photonic crystal
fiber-based SPR sensor for cancer detection utilizing hybrid nanocomposite
layers. Plasmonics.

[77] Sudheer V., Kumar S. S., Sankararaman S. (2020). Ultrahigh
sensitivity surface plasmon resonance-based fiber-optic sensors using
metal-graphene layers with Ti3C2Tx MXene overlayers. Plasmonics.

[78] Sudheer V., SarathKumar S., Sankararaman S. (2021). Nanostructured
ZnO and ZnO: Pd with MXene overlayer SPR biosensors. Opt. Quantum Electron..

[79] Vikas, Verma R. (2021). On the application of few layer Ti_3_C_2_ MXene on fiber optic SPR sensor for performance
enhancement. Eur. Phys. J. D.

[80] Verma V. K., Kumar R., Pal S., Prajapati Y. K. (2022). Highly sensitive MXene-immobilized long range SPR sensor
for biomolecule detection. Opt. Mater..

[81] Mumtaz F., Roman M., Zhang B., Abbas L. G., Dai Y., Ashraf M. A., Fiaz M. A., Kumar A. (2022). MXene (Ti3C2Tx) coated
highly-sensitive D-shaped photonic crystal fiber based SPR-biosensor. Photon. Nanostruct: Fundam. Appl..

[82] Kaur B., Kumar S., Kaushik B. K. (2022). MXenes-based fiber-optic SPR sensor
for colorectal cancer diagnosis. IEEE Sens.
J..

[83] Wu Y., Shen T., Feng Y., Liu C., Liu X., Wang S. (2022). PCF sensor coated with Au-graphene/MXene
for a low refractive index and a wide detection range. J. Opt. Soc. Am. B.

[84] Kumar A., Verma P., Jindal P. (2022). Surface plasmon resonance biosensor
based on a D-shaped photonic crystal fiber using Ti3C2Tx MXene material. Opt. Mater..

[85] Mu R., Wan H., Shi W., Liang H., Lou Y. (2023). Design and theoretical analysis of
high-sensitive surface plasmon resonance sensor based on Au/Ti 3C2Tx-MXene
hybrid layered D-shaped photonic crystal fiber. IEEE Sens. J..

[86] Srivastava R., Pal S., Prajapati Y. K. (2023). MXene-assisted
D-shaped photonic crystal fiber probe with high sensitivity for detection
of tuberculosis. Plasmonics.

[87] Li K., Yin Z., Wang C., Li S. (2024). Enhancement of SPR effect and sensing characteristics in photonic
crystal fiber with Ti 3 C 2 T x-Mxene/silver film. J. Lightwave Technol..

[88] Zhou Y., Yan X. (2022). D-shaped fiber surface plasmon resonance
refractive index sensor enhanced by MXene (Ti3C2Tx). IEEE Photonics J..

[89] Zhu J., Zhao C., Xia B., Wang N., Chen X., Jing X., Chen M., Xu X. (2024). An enhanced SPR optical
fiber biosensor using Ti 3 C 2 T x MXene/AuNPs for label-free and
sensitive detection of human IgG. Nanoscale.

[90] Zhao C., Zhu J., Ma X., Sun J., Wang N., Chen X., Chen X., Wang Y., Wang C., Jing X. (2025). High sensitive optical fiber SPR
biosensor enhanced by Ti_3_C_2_T_x_ MXene
for label-free detecting Staphylococcus aureus. Microchem. J..

[91] Mohd Makhfuz M. J., Yusoff N., Ahmad H. (2025). Ti2C MXene-based
arc-shaped fiber SPR sensor for refractive index sensing. Opt. Fiber Technol..

[92] Zhang Y., Ding L., Xiao B., Wang S., Meng W., Gao L., Che T., Zheng X. (2025). Ti3C2MXene/GNRs for synergistically highly enhanced sensitivity of
optical fiber SPR acetylcholine biosensors via an electrostatic layer-by-layer
assembly method. Biosens. Bioelectron..

[93] Huang Y., Chen Y., Yuan P., Luo B., Wu S., Shi S., Zhao M. (2025). Ultra-sensitive SPR fiber-optic biosensor
based on MNM structure with Ti_3_C_2_ MXene/PDA
modification for placental growth factor detection. Sens. Actuators, B.

[94] Li H., Huang T., Yuan H., Lu L., Cao Z., Zhang L., Yang Y., Yu B., Wang H. (2023). Combined ultrasensitive detection of renal cancer proteins and cells
using an optical microfiber functionalized with Ti3C2MXene and gold
nanorod-nanosensitized interfaces. Anal. Chem..

[95] Li G., Singh R., Guo J., Zhang B., Kumar S. (2023). Nb2CTx MXene-assisted double S-tapered
fiber-based LSPR sensor with improved features for tyramine detection. Appl. Phys. Lett..

[96] Liu X., Singh R., Li M., Li G., Min R., Marques C., Zhang B., Kumar S. (2023). Plasmonic
sensor based on offset-splicing and waist-expanded taper using multicore
fiber for detection of Aflatoxins B1 in critical sectors. Opt. Express.

[97] Zhang W., Singh R., Liu F.-Z., Marques C., Zhang B., Kumar S. (2023). WaveFlex biosensor: a flexible-shaped plasmonic optical fiber sensor
for histamine detection. IEEE Sens. J..

[98] Li X., Singh R., Kumar K., Zhang B., Guo J., Kumar S., Li G. (2024). Fe_3_O_4_-Chitosan/MXene-Assisted MMF-MCF-MMF-based WaveFlex
Biosensor with Improved Features for Doxorubicin Detection. IEEE Sens. J..

[99] Lang X., Singh R., Zhang B., Kumar S. (2024). Highly sensitive
TIT4T fiber-based WaveFlex biosensors functionalized with MXene-QDs
for xanthine detection. IEEE Sens. J..

[100] Singh R., Zhang W., Liu X., Zhang B., Kumar S. (2024). WaveFlex biosensor: MXene-immobilized
W-shaped fiber-based LSPR sensor for highly selective tyramine detection. Opt. Laser Technol..

[101] Liu C., Wang R., Shao Y., Chen C., Wu P., Wei Y., Gao Y. (2021). Detection
of GDF11 by using a Ti3C2-MXene-based fiber SPR biosensor. Opt. Express.

[102] Yang W., Cheng Y., Jiang M., Jiang S., Liu R., Lu J., Du L., Li P., Wang C. (2022). Design and fabrication
of an ultra-sensitive Ta2C MXene/Au-coated tilted grating sensor. Sens. Actuators, B.

[103] Liu R., Yang W., Lu J., Shafi M., Jiang M., Jiang S. (2023). Plasmonic optical fiber
gratings based on few-layer Ta2C MXenes for refractive index sensing. Nanotechnology.

[104] Ahmad H., Mohd Makhfuz M. J., Yusoff N., Zakaria R. (2024). The effect of Ti2C
MXene on the performance of optical fiber-based surface plasmon resonance
sensor towards lead detection. Mater. Sci. Eng.
B.

[105] Furen Y., Hongyu S., Shuxian G., Yanpei X., Qi W. (2025). Optical Fiber Surface Plasmon Resonance
Alcohol Sensor Based on MXene/S n O 2/Au Structure. Plasmonics.

[106] Li W., Miao Y., Guo T., Zhang K., Yao J. (2021). Nb2CTx MXene-tilted fiber Bragg grating optofluidic system based
on photothermal spectroscopy for pesticide detection. Biomed. Opt. Express.

[107] Guntner A. T., Abegg S., Konigstein K., Gerber P. A., Schmidt-Trucksass A., Pratsinis S. E. (2019). Breath sensors for health monitoring. ACS Sens..

[108] Zhu C., Gerald R. E., Huang J. (2021). Metal-organic framework
materials coupled to optical fibers for chemical sensing: A review. IEEE Sens. J..

[109] Dutta T., Noushin T., Tabassum S., Mishra S. K. (2023). Road map
of semiconductor metal-oxide-based sensors: a review. Sensors.

[110] Chidambaram A., Stylianou K. C. (2018). Electronic
metal-organic framework sensors. Inorg. Chem.
Front..

[111] Zhao H., Tan X., Chai H., Hu L., Li H., Qu L., Zhang X., Zhang G. (2025). Recent advances
in conductive MOF-based electrochemical sensors. Chin. Chem. Lett..

[112] Habib T., Zhao X., Shah S. A., Chen Y., Sun W., An H., Lutkenhaus J. L., Radovic M., Green M. J. (2019). Oxidation stability of Ti3C2Tx MXene
nanosheets in solvents and composite films. npj 2D Mater. Appl..

[113] Lee Y., Kim S. J., Kim Y.-J., Lim Y., Chae Y., Lee B.-J., Kim Y.-T., Han H., Gogotsi Y., Ahn C. W. (2020). Oxidation-resistant titanium carbide
MXene films. J. Mater. Chem. A.

[114] Zhao X., Holta D. E., Tan Z., Oh J.-H., Echols I. J., Anas M., Cao H., Lutkenhaus J. L., Radovic M., Green M. J. (2020). Annealed Ti3C2Tz
MXene films for oxidation-resistant functional coatings. ACS Appl. Nano Mater..

[115] Yan L., Luo X., Yang R., Dai F., Zhu D., Bai J., Zhang L., Lei H. (2022). Highly thermoelectric ZnO@ MXene
(Ti3C2T x) composite films grown by atomic layer deposition. ACS Appl. Mater. Interfaces.

[116] Zheng H., Xu L., Yan Q., Liu Z., Chen H., Lian H., Chen Y., Fei T., Hu Y., Xue F. (2025). Additive-Free Ti3C2Tx MXene Actuator with Large
Deformation, Programmability, and High-Humidity Stability via Precise
Interlayer Spacing Control Engineering. Adv.
Sci..

[117] Zhang Y. Z., Wang Y., Jiang Q., El-Demellawi J. K., Kim H., Alshareef H. N. (2020). MXene printing and patterned coating for device applications. Adv. Mater..

[118] Younis A., Idrisov E., Thaker S., Hamed F., Sadki E. H., Iqbal M. Z., Mahmood T., Shabbir B., Bao Q. (2025). Advances in MXene-Based Electronics
via Surface and Structural Redesigning and Beyond. Adv. Electron. Mater..

[119] Xu S., Zhang X., Zheng T., Zhao Z., Shang C., Hu Z., Dong M., Qiao Y., Bai C., Zhang X. (2025). Advancements
in high-performance MXene composite fibers integrated with various
functional materials: Fabrication, functionalization, property enhancement,
and applications. J. Mater. Sci. Technol..

